# Blood biochemical landscape and new insights into clinical decision-making for polycystic ovary syndrome in Chinese women: a prospective cohort study

**DOI:** 10.3389/fendo.2025.1534733

**Published:** 2025-05-01

**Authors:** Yutong Li, Xiufeng Lin, Ke Zou, Jing Du, Qingni Li, Linkun Zhong, Shan Jiang

**Affiliations:** ^1^ The First Clinical College, Guangdong Medical University, Zhanjiang, Guangdong, China; ^2^ Department of General Surgery, Zhongshan City People’s Hospital, Zhongshan, Guangdong, China; ^3^ Reproductive Center, Boai Hospital of Zhongshan, Zhongshan, Guangdong, China

**Keywords:** cryptotanshinone, glucosaminyl (N-acetyl) transferase 2, SHBG, polycystic ovary syndrome, Mendelian randomization (MR)

## Abstract

**Introduction:**

The Polycystic ovary syndrome (PCOS), a prevalent endocrine disorder affecting women’s reproductive and metabolic health, faces diagnostic challenges due to heterogeneous clinical presentations and the absence of reliable biomarkers. This study investigates the role of Glucosaminyl (N-acetyl) transferase 2 (GCNT2) in modulating sex hormone-binding globulin (SHBG) and its potential as a therapeutic target in PCOS pathophysiology.

**Methods:**

A prospective cohort of 103 PCOS patients treated with oral contraceptives (2021–2024) was established. Bidirectional Mendelian randomization (MR) was employed to assess genetic associations and causal relationships between PCOS and SHBG. Molecular docking studies evaluated cryptotanshinone’s binding affinity to key proteins (COL1A1, COL4A2, COL6A2) in the PI3K/Akt pathway. GCNT2’s regulatory effects on collagen synthesis and extracellular matrix pathways. Pharmacokinetic profiling validated therapeutic viability.

**Results:**

Bidirectional MR revealed significant genetic associations (*P* < 0.001) and causal links between PCOS and SHBG, implicating GCNT2 as a key modulator. Cryptotanshinone exhibited strong binding affinity to PI3K/Akt signaling pathway proteins and favorable pharmacokinetic properties. Enrichment analyses highlighted GCNT2’s role in collagen biosynthesis (*FDR* < 0.05) and extracellular matrix regulation.

**Discussion:**

This study identifies GCNT2 as a critical mediator of PCOS pathophysiology through SHBG modulation and collagen remodeling. Cryptotanshinone emerges as a promising therapeutic candidate, targeting PI3K/Akt signaling pathway with high specificity. These findings advance the understanding of PCOS mechanisms and provide a foundation for biomarker-driven diagnostics and precision therapeutics. Further validation in clinical trials is warranted to translate these insights into practice.

## 1 Introduction

Polycystic ovary syndrome (PCOS) is the most common endocrine disorder affecting women of reproductive age globally, with a prevalence of up to 33% (1, 2). According to recent studies, there are approximately 1.55 million cases of PCOS among women worldwide (3). The characteristics of PCOS include hyperandrogenism, hyperinsulinemia, and disrupted adipokine secretion, with patients often experiencing β-cell dysfunction (4). Low birth weight and fetal exposure to androgens may contribute to the development of PCOS phenotypes, predisposing individuals to metabolic complications. PCOS poses the multifaceted challenge for patients and healthcare providers, with emerging evidence indicating that its metabolic impact extends beyond reproductive symptoms. PCOS is closely associated with insulin resistance, affecting nearly 70% of patients, which contributes to hyperinsulinemia and widespread metabolic dysregulation. These disturbances markedly increase the risk of type 2 diabetes, cardiovascular diseases, and metabolic syndrome in affected women, highlighting the urgent need for targeted and effective therapeutic strategies (5, 6). Consequently, PCOS presents multifaceted challenges for both patients and healthcare providers (7, 8). As research advances in unraveling the molecular mechanisms of PCOS, it is evident that a comprehensive understanding of its pathophysiology is crucial for designing precise and effective therapeutic interventions.

Glucosaminyl (N-acetyl) transferase 2 (GCNT2) is an essential glycosyltransferase enzyme that mediates the biosynthesis of complex carbohydrates, particularly through the modification of glycoproteins and glycolipids. It catalyzes the transfer of N-acetylglucosamine residues to specific substrates, thereby modulating the structural and functional characteristics of glycoproteins, which are integral to processes such as cellular signaling, adhesion, and immune regulation (9). The glycosylation patterns mediated by GCNT2 are essential for protein functionality, influencing their stability, cellular localization, and molecular interactions. Notably, GCNT2 has been identified as a key regulator of sex hormone-binding globulin (SHBG), a glycoprotein that modulates the bioavailability of sex hormones, including testosterone and estradiol, thereby playing a critical role in hormonal homeostasis (8, 10). Dysregulation of SHBG is associated with several endocrine disorders, including PCOS, which is characterized by hormonal imbalances and metabolic dysfunction (2, 11, 12). Studies have indicated that alterations in GCNT2 expression can lead to changes in SHBG levels, further implicating GCNT2 in the pathophysiology of PCOS and its associated symptoms, such as insulin resistance and infertility (13). Furthermore, GCNT2 has been shown to play a role in the PI3K/Akt pathway, which is crucial for cell survival, proliferation, and metabolism (14, 15). Investigating the specific mechanisms through which GCNT2 influences SHBG levels and other key pathways could provide new insights into the development of targeted therapies for PCOS and related conditions.

This study is designed to address three key objectives: (1) the identification of novel blood-based diagnostic biomarkers for PCOS through comprehensive analysis of clinical blood biochemical indicators, with a specific focus on the regulatory role of GCNT2, and the evaluation of their diagnostic precision and clinical applicability, (2) the investigation of the effects of estradiol-cyproterone acetate (EE-CA) oral contraceptives on serum SHBG levels in PCOS patients, with an emphasis on potential interactions with GCNT2 expression, and (3) the discovery of candidate genes, particularly GCNT2, for early detection and targeted therapeutic interventions in PCOS. To this end, we have established a well-defined training cohort comprising 103 PCOS patients, systematically analyzing their blood biochemical profiles and employing advanced machine learning techniques to assess the specificity and predictive capacity of these indicators for PCOS diagnosis. Bidirectional two-sample Mendelian randomization (MR) will be utilized to explore risk associations of target proteins, including GCNT2, based on identified blood biochemical markers, aiming to uncover potential therapeutic targets and elucidate their molecular mechanisms. Additionally, we will leverage AI-Driven Drug Design and computational biology approaches to develop low-toxicity, high-efficacy lead compound targeting GCNT2 and associated proteins, thereby paving the way for personalized and precision-based treatment strategies for PCOS patients.

## 2 Methods

### 2.1 Materials and sources of clinical samples

Blood samples were collected from 103 PCOS patients receiving treatment with Diane-35 at Zhongshan Boai Hospital (Third Class, Category A). Diane-35, produced by Bayer Weimar GmbH & Co. KG (Bayer, National Drug Approval Number HJ20170210, Germany), was administered at a dosage of one tablet per day. Each tablet contains a combination of EE-CA and ethinylestradiol, which are essential components for managing the symptoms associated with PCOS; Insulin (INS) measurement kit (YHLO, Shenzhen, China); D-dimer measurement kit (PUSHKANG, Hunan, China); Elecsys Müller Tube (AMH) Hormone Plu (Roche Diagnostics, USA); Elecsys SHBG (Roche Diagnostics, USA); Access TSH (3^rd^ IS) (Beckman Coulter, USA); Access hFSH (Beckman Coulter, USA); Access hLH (Beckman Coulter, USA); Access Testosterone (TESTO) (Beckman Coulter, USA); Total Cholesterol (CHOL) Assay Kit (Gcell, Beijing, China); High-Density Lipoprotein Cholesterol (HDL-C) Assay Kit (Gcell, Beijing, China); Low-Density Lipoprotein Cholesterol (LDL-C) Assay Kit (Gcell, Beijing, China); Triglycerides (TG) Assay Kit (Gcell, Beijing, China); Glucose Kit (GLU) (Medicalsystem, Zhejiang, China); Homocysteine (Hcy, HCY) Determination Reagent Kit (ERKN, Zhejiang, China).

### 2.2 Detection instruments

Fully automated coagulation analyzer (Sysmex Europe, CS5 100, Kobe, Japan); fully automated biochemical immunoassay system (Roche Diagnostics, COBAS^®^ 8000, USA); fully automated chemiluminescent immunoassay analyzer (Beckman Coulter, UniCel DxI 800 Access, USA); fully automated biochemical analyzer (Beckman Coulter, AU5831/AU5821, USA); chemiluminescence detector (YHLO, iFlash 3000-A, Hunan, China).

### 2.3 Study cohort construction

The study included 103 PCOS patients who visited our hospital between March 2021 and July 2024. These patients primarily presented with oligomenorrhea or amenorrhea, dysfunctional uterine bleeding, or hyperandrogenemia. Type A PCOS patients typically exhibit more pronounced metabolic alterations and represent the most prevalent subtype (16). In this study, all 103 enrolled patients were newly diagnosed with Type A PCOS.

PCOS is diagnosed based on the Rotterdam Consensus criteria when at least two of the following three conditions are met: menstrual irregularities (oligomenorrhea or amenorrhea), hyperandrogenism (clinically or biochemically confirmed), and specific ovarian characteristics (at least 12 follicles of 2-9 mm in each ovary or ovarian volume >10 ml). This method facilitates accurate diagnosis and informs subsequent clinical management (17). Exclusion criteria comprised individuals who had used hormonal medications, vitamins, metformin, or homocysteine-elevating agents within the preceding six months, as well as those with documented deficiencies in folate, vitamin B12, or vitamin B6.

Patients’ weight and height were recorded to calculate the body mass index (BMI = weight (kg)/height (m)²). A BMI of 25.1-30 kg/m² indicated overweight, while a BMI above 30 kg/m² signified obesity. Waist and hip circumferences were measured at the narrowest and widest points, respectively. The waist-to-hip ratio (WHR) was then calculated, with a WHR > 0.72 considered abnormal. All patients took a combined oral contraceptive containing 35 μg of EE and 2 mg of CA (Diane-35, Bayer, Germany) for 21 days each month, followed by a 7-day drug-free interval, for a total of three cycles. Assessments were performed before treatment and following the completion of the third cycle, evaluating various parameters such as hormone levels (including follicle-stimulating hormone [FSH], luteinizing hormone [LH], TESTO, and free testosterone [FAI]), lipid profiles (CHOL, LDL-C, HDL-C and TG), as well as homocysteine, folate, and serum insulin levels. Blood samples were obtained during the early follicular phase (days 3-5) of spontaneous or progesterone-induced bleeding, collected between 8:00 and 9:00 AM after an overnight fast. The samples were kept on ice, promptly centrifuged, and the serum was stored at -80°C for future analysis.

### 2.4 Detection of biochemical markers and metabolic indicators

INS levels were measured using a chemiluminescence detector YHLO iFlash 3000-A. D-dimer was quantified via the PUSHKANG latex immunoturbidimetric method. AMH and SHBG were assessed using the Roche COBAS^®^ 8000 system, employing electrochemiluminescence. The levels of TSH, FSH, LH and TESTO were measured using the Beckman Coulter UniCel DxI 800 Access system, which utilizes chemiluminescent assays. GLU levels were determined using the Meikang Biotechnology kit, while HCY was measured via the enzyme cycle method with the ERKN kit. All analyses were performed using fully automated systems to ensure accuracy and efficiency in the laboratory. CHOL, HDL-C, LDL-C, TG and transaminases were analyzed with the Beckman Coulter AU5831/AU5821 biochemical analyzers.

### 2.5 Selection of diagnostic and therapeutic predictive markers for PCOS using machine learning and decision models

Statistical analysis of clinical characteristics, biochemical markers, and metabolic features of PCOS patients was performed using R 4.3.2, focusing on indicators that significantly changed following EE-CA treatment (*P* < 0.05). LASSO regression and random forest (RF) algorithms were employed to train a cohort of 103 PCOS patients, facilitating the selection of biomarkers for diagnosis and treatment outcome prediction. Additionally, a decision curve analysis (DCA) was conducted to compare the predictive efficacy of these novel biomarkers against established clinical and biochemical markers [LH/FSH, TESTO, and FAI [FAI = 100 × (TESTO/SHBG)] (18)], thereby aiding in the identification and validation of new biomarkers for PCOS.

### 2.6 Data source

**Figure 1:**
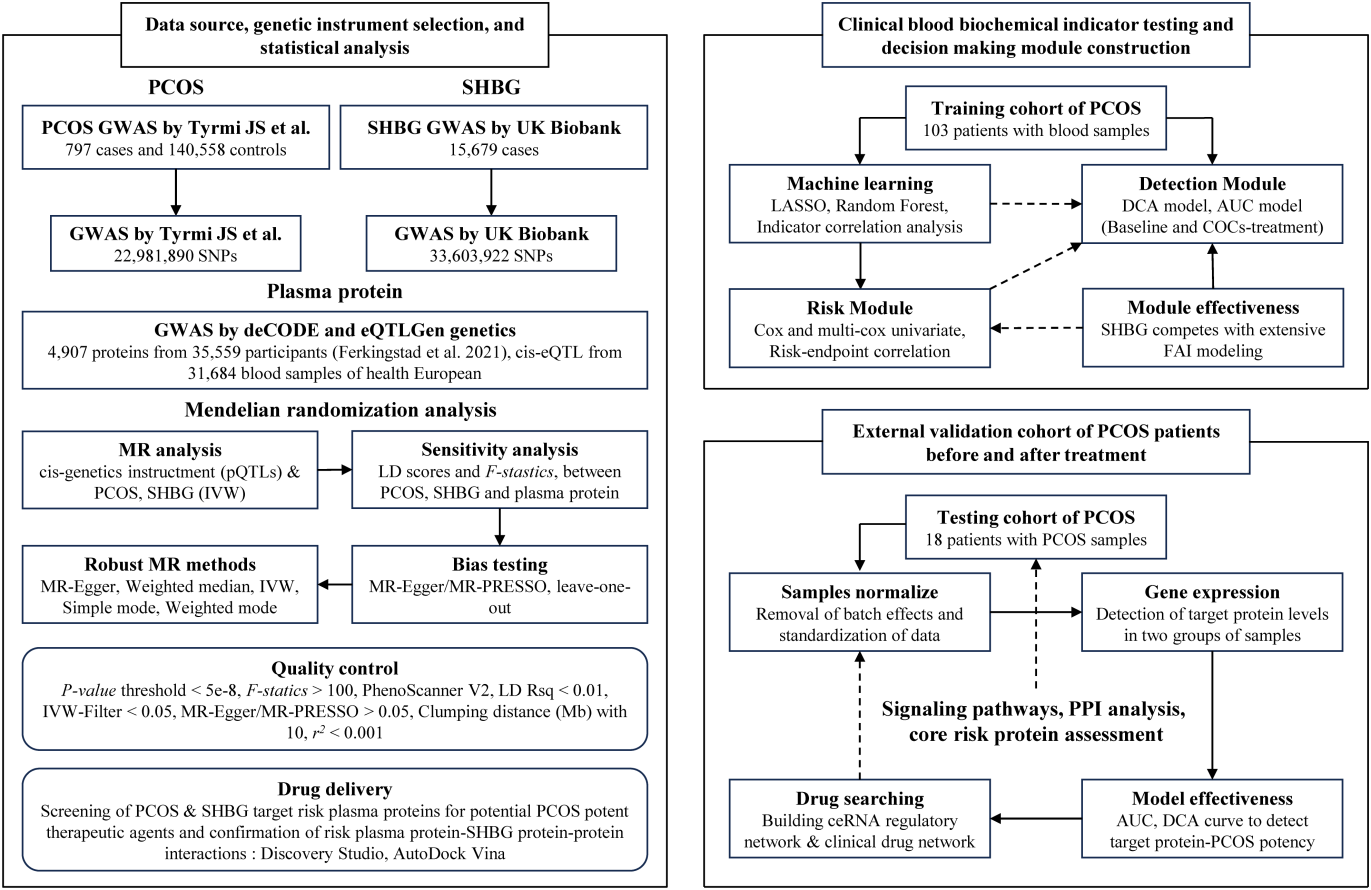
The workflow diagram and study design of this research.

The overview of data sources, genetic instrument selection, and statistical analysis in this study is illustrated in **Figure 1**. The summary data for PCOS was obtained from a large genome-wide association study (GWAS) conducted by Tyrmi JS et al., which included 797 cases and 140,558 controls (19). The summary data for SHBG was obtained from the UK Biobank, comprising serum SHBG levels from up to 189,473 women of European ancestry (20). PCOS participants were diagnosed based on the criteria established by the National Institutes of Health, the Rotterdam criteria, or self-reported diagnoses (21).

Plasma protein quantitative trait loci (pQTL) data were obtained from a comprehensive integrative study conducted by Ferkingstad et al., which detailed pQTL information for 4,907 plasma proteins from 35,559 Icelandic participants (22). To pinpoint cis-pQTLs, we established the following criteria: (1) pQTLs must demonstrate genome-wide significance (*P* < 5×10^−8^); (2) pQTLs should be situated outside the major histocompatibility complex region; (3) independence is required, defined by linkage disequilibrium (LD) with *r²* < 0.001; (4) pQTLs must show cis-effects, meaning they fall within a 1000 kb range of the corresponding protein-coding sequences; (5) pQTLs should not be considered weak instruments (*F-statistic* > 100); and (6) Single nucleotide polymorphisms (SNPs) that were palindromic or had missing data were excluded. In total, we identified 6,238 cis-acting SNPs linked to 1,729 proteins. To further validate our findings, we obtained blood expression quantitative trait locus (eQTL) datasets from eQTLGen (https://www.eqtlgen.org/). This dataset includes cis-eQTL information for 16,987 genes derived from 31,684 blood samples of European ancestry (19).

The independent validation cohort for PCOS patients was sourced from the GEO database (https://www.ncbi.nlm.nih.gov/), specifically GSE87435 dataset, which includes 36 individuals diagnosed with PCOS. Molecular 3D structures of plasma proteins and natural compounds were retrieved from the UniProt (https://www.uniprot.org/), RCSB PDB (https://www.rcsb.org/), and PubChem (https://pubchem.ncbi.nlm.nih.gov/). All data utilized in this study were derived from existing publications or publicly available databases that had obtained ethical approval and informed consent, thus negating the need for additional ethical clearance.

### 2.7 MR analysis

Plasma proteins were treated as exposures, with SHBG and PCOS as outcomes. When a plasma protein had a single pQTL as an instrumental variable (IV), we assessed the causal effect using the Wald ratio (WR) method (23). In instances with two or more IVs, we employed inverse variance weighting (IVW) (24). Statistical results were expressed as odds ratios (OR) with 95% confidence intervals (95% CI), using a nominal significance threshold of *P* < 0.05. To minimize false positives, a false discovery rate (FDR) correction was applied, defining significant findings as those with *FDR* < 0.05. If a protein showed a nominally significant association that lost significance after correction, it was classified as suggestively significant.

MR analyses were conducted using the “TwoSampleMR” package within R 4.3.2. To assess the robustness of our findings, we utilized Cochran’s Q test and the MR-Egger intercept test to evaluate potential heterogeneity and horizontal pleiotropy after applying the IVW method (with *P* > 0.05 indicating the absence of these issues) (25). For the IVW analysis, both fixed-effect and random-effect models were used; the fixed-effect model applied when no significant heterogeneity was detected, while the random-effect model was used in its presence.

We employed MR-PRESSO to identify outliers, specifically potential pleiotropic SNPs, and to estimate causal effects after their exclusion (26). Leave-one-out analysis was conducted to determine if influential SNPs drove the causal effect, recalculating MR estimates by omitting one instrument at a time (27). Additionally, using the “phenoscanner” tool, we performed phenotype scanning to assess whether the identified pQTLs were associated with other traits and exhibited pleiotropic effects (28, 29). pQTLs were considered pleiotropic if they met two criteria: (1) associations that achieved genome-wide significance (*P* < 5 × 10^−8^); (2) known associations with established risk factors for SHBG and PCOS.

### 2.8 Functional enrichment analysis of target proteins and their regulation in PCOS

We constructed a receiver operating characteristic (ROC) model using a cohort of PCOS patients from the GEO database to evaluate the diagnostic efficacy of potential risk plasma proteins related to SHBG and PCOS. We examined the differential mRNA expression levels of risk plasma proteins in PCOS versus normal ovarian tissues, identifying those with the greatest contribution to PCOS risk for further analysis, including genome-wide co-expression gene analysis (*logFC* > 1, *P* < 0.05). Protein-protein interaction (PPI) networks were constructed using the STRING platform (https://string-db.org/), and chromosomal co-localization analysis was performed for the identified risk proteins. Gene Ontology (GO) and Kyoto Encyclopedia of Genes and Genomes (KEGG) analyses were conducted to elucidate the molecular mechanisms through which the target plasma proteins regulate processes in PCOS. All analyses and visualizations were carried out using R 4.3.2.

### 2.9 High-throughput virtual screening (HTVS) of natural compounds targeting the identified plasma proteins

Natural compounds potentially targeting the identified plasma proteins were sourced from the TCM-Suit, HERB, and TCMSID database (http://tcm-suite.aimicrobiome.cn/, http://herb.ac.cn/, https://tcm.scbdd.com/). These compounds were imported into the Discovery Studio 2019 (DS) for molecular conformation expansion. GCNT2, identified as the most promising plasma protein biomarker for PCOS, had its 3D structure imported into DS. The protein amino acid sequence was imported into SWISS-MODEL to construct the protein crystal structure in a human physiological environment (30). We performed protein residue modifications and active pocket predictions under the CHARMM36 force field in a human aqueous environment, conducting both rapid and precise molecular docking. The docking energies and intermolecular interaction energies were calculated, allowing us to select the optimal conformation for each compound. The best conformations were then visualized using PyMOL to generate 3D interaction models. Additionally, we validated the results from DS using AutoDock Vina, calculating the protein-ligand binding energies through Vina docking.

### 2.10 Analysis of the pharmacokinetic and toxicological properties of lead compounds

The structures of lead compounds processed in DS were imported into Swiss-ADME (http://www.swissadme.ch/), VenomPred 2.0 (31) and VirtualTaste (https://insilico-cyp.charite.de/) to evaluate their ADME, toxicokinetic (including hepatotoxicity, neurotoxicity, mutagenicity, hormonal toxicity, strong irritancy, and acute oral toxicity), and palatability analysis. Subsequently, the collected data were visualized using R 4.1.3.

### 2.11 The fast fourier transform algorithm supports protein-protein molecular docking

We employed a protein-protein docking technique based on the FFT algorithm, implemented through DS. This approach leverages the mathematical properties of FFT to convert the spatial coordinates of protein structures into frequency space, facilitating efficient searches for optimal binding orientations between protein complexes. Specifically, the FFT algorithm processes molecular surface interactions in the frequency domain, enabling rapid identification of potential binding sites while significantly reducing computational time and maintaining high accuracy in predicting protein interactions. In implementation process, we first constructed three-dimensional models for both the target protein and the ligand protein, converting them into a format suitable for FFT calculations. Subsequently, we discretized the molecular surfaces to construct an interaction matrix and performed convolution operations in the frequency domain to filter out the binding modes with the lowest energy from a vast array of possible conformations. Finally, the generated docking models were validated using energy scoring functions and further refined through molecular dynamics simulations.

## 3 Results

### 3.1 Analysis of the correlation between clinical and hematological indicators in patients with PCOS before and after treatment

All participating women completed the study, with each patient exhibiting clear signs of PCOS during ultrasound examinations. Among the patients, 22 were found to have folate metabolism disorders (21.4%), while 27 experienced adverse outcomes following artificial embryo transfer (IUI/IVF) (26.2%). 19 patients encountered pregnancy complications (18.4%), 13 presented with signs of hyperandrogenism (12.6%), and 10 had hyperinsulinemia (9.7%). 10 patients exhibited hypercholesterolemia (9.7%), and 9 were diagnosed with hyperhomocysteinemia (HHCY) (8.7%). Following treatment with oral contraceptives, patients exhibited significant improvements in serum levels of LH, SHBG, HCY, LH/FSH ratio, and TESTO (*P* < 0.05). Notably, SHBG emerged as a promising novel biomarker for assessing the diagnosis and treatment efficacy in PCOS patients (*P* < 0.0001) (**Table 1**).

**Table 1:** Endocrinological and metabolic characteristics of PCOS subjects at baseline and post-treatment (n = 103).

	Baseline	Post-treatment	*P-value*
Age (year)	29.44 ± 3.74	/	/
WHR	0.85 ± 0.06	/	/
D-D (mg/L FEU)	0.40 ± 1.18	/	/
AMH (ng/ml)	7.19 ± 4.28	/	/
HDL-C	1.46 ± 0.31	/	/
LDL-C	3.27 ± 0.70	/	/
TSH (mU/L)	2.14 ± 1.38	/	/
CHOL (mmol/L)	5.13 ± 0.80	/	/
Systolic	114.50 ± 10.43	/	/
Diastolic	77.10 ± 7.69	/	/
BMI (kg/m^2^)	24.80 ± 3.64	24.37 ± 3.72	NS
FSH (mIU/ml)	6.47 ± 1.28	6.40 ± 1.55	NS
LH (mIU/ml)	9.53 ± 5.44	7.43 ± 3.28	0.0018
GLU (mmol/L)	5.43 ± 0.58	5.28 ± 0.50	NS
SHBG	41.36 ± 26.70	114.02 ± 57.43	< 0.0001
INS (IU/ml)	16.66 ± 9.76	17.31 ± 8.24	NS
TG (mmol/L)	1.46 ± 0.94	/	/
HCY (μmol/L)	9.88 ± 2.11	8.92 ± 1.84	0.0015
TESTO (ng/ml)	0.72 ± 0.26	0.62 ± 0.23	0.0025
LH/FSH	1.48 ± 0.81	1.22 ± 0.63	0.0167

### 3.2 Changes in clinical and hematological indicators in PCOS patients before and after treatment with oral contraceptives

Although only 32 patients (31.1%) had a waist circumference greater than 88 cm, central obesity, as estimated by the WHR, was observed in 13 patients (40.6%). Among the participants, 35 had normal weight (34%), while 64 were classified as obese (62.1%). Throughout the treatment period, no significant changes were noted in BMI or WHR. All patients maintained normal levels of vitamin B12 and folate at both the beginning and end of the study. During treatment, TESTO levels decreased from 0.72 ± 0.26 to 0.62 ± 0.23 ng/ml (*P* = 0.0025), and the FAI dropped significantly from 11.7 ± 4.9 to 4.7 ± 3.6 (*P* < 0.0001). In contrast, SHBG) levels significantly increased from 41.36 ± 26.70 to 114.02 ± 57.43 (*P* < 0.0001). Additionally, the levels of HCY and the LH/FSH ratio showed substantial improvement, decreasing from 9.88 ± 2.11 to 8.92 ± 1.84 (*P* = 0.0015) and from 1.48 ± 0.81 to 1.22 ± 0.63 (*P* = 0.0167), respectively. The data indicate that oral contraceptive treatment significantly improved SHBG levels in both obese and non-obese patients, with values rising from 34.09 ± 24.40 to 115.35 ± 55.56 and from 94.90 ± 25.47 to 132.73 ± 67.28 (*P* < 0.0001) (**Table 1**). Pearson and Spearman correlation analyses revealed a strong association between SHBG levels before and after treatment and factors such as BMI, TESTO, GLU, INS, and HCY (*R* > 0.2, *P* < 0.05) (**Supplementary Tables S1, S2**).

### 3.3 Machine learning screening of PCOS-specific diagnostic and treatment efficacy assessment indicators

**Figure 2:**
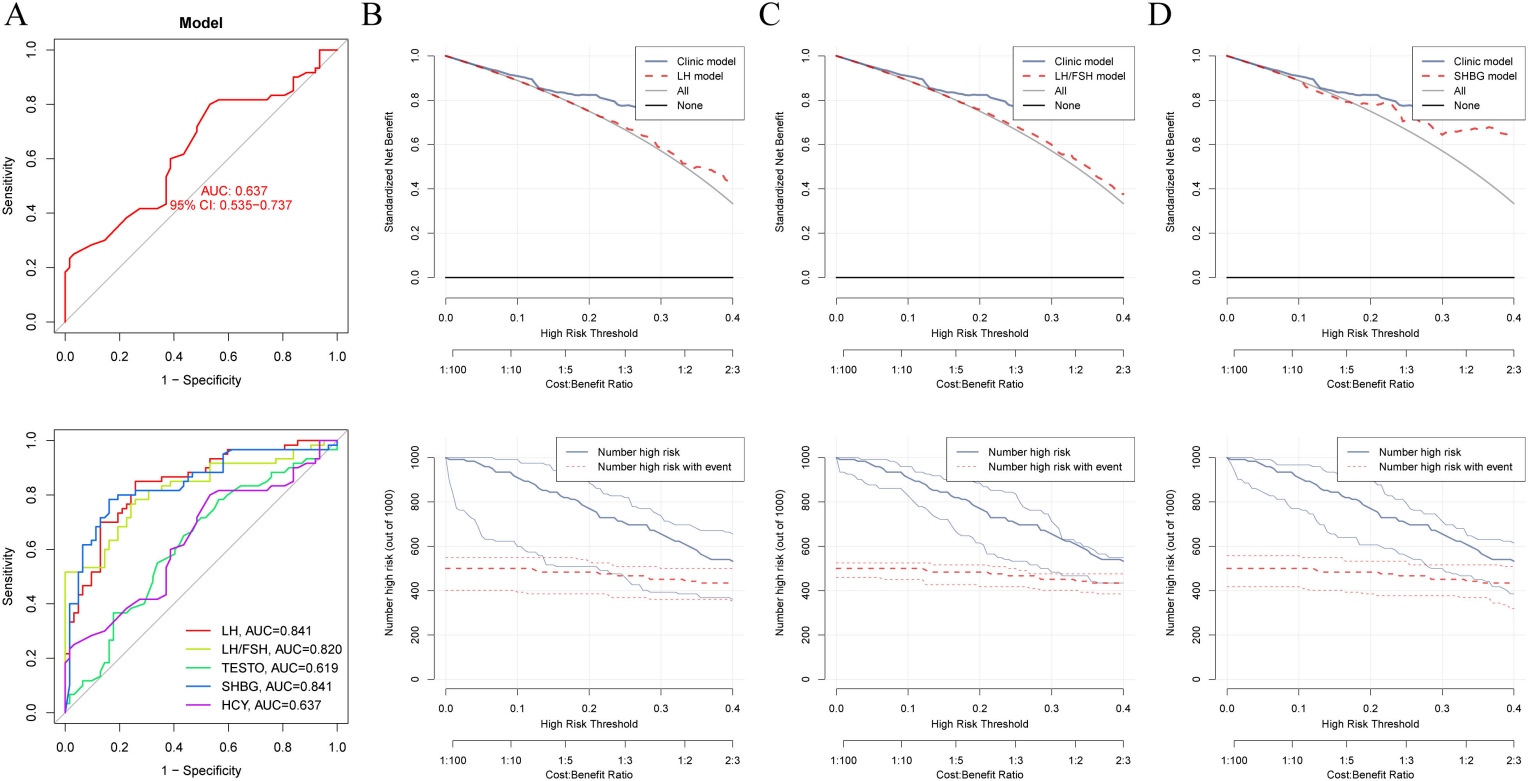
Machine learning and decision curve analysis for the identification of novel biomarkers for PCOS. **(A)**. The AUC curve evaluates the specificity and diagnostic efficacy of potential biomarkers for PCOS, including LH, LH/FSH ratio, TESTO, HCY, and SHBG. **(B–D)**. Comparison of the decision-making capabilities of LH, LH/FSH, and SHBG with clinical model indicators, along with their contribution to the prognostic risk curve for PCOS.

**Figure 3:**
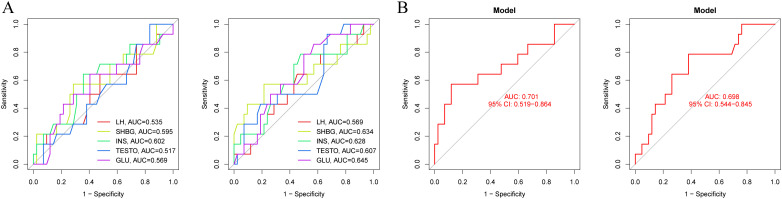
Diagnostic models of metabolic biomarkers before and after oral contraceptive treatment in PCOS patients. **(A)** AUC diagnostic specificity curves for metabolic characteristics in PCOS patients before and after treatment. **(B)** Specific AUC curves for PCOS in the two models before and after treatment.

The combined analysis using LASSO and RF identified LH, LH/FSH ratio, TESTO, SHBG, and HCY as potential specific metabolic markers for PCOS, with AUC values of 0.841, 0.820, 0.619, 0.841, and 0.637, respectively. Our clinical decision model demonstrates a reliable capacity to assess treatment efficacy in PCOS patients, achieving an AUC of 0.637 (95% CI: 0.535-0.737) (**Figures 2A**, **S1A, B**). With an AUC greater than 0.7 indicating high specificity for PCOS, LH, LH/FSH ratio, and SHBG are considered the most promising potential biomarkers for the condition (**Figures 2B–D**). Model training on the cohort of PCOS patients before and after oral contraceptive treatment was conducted to assess the diagnostic efficacy of blood biochemical markers. The results indicated that prior to treatment, only INS demonstrated diagnostic specificity for PCOS (AUC = 0.602). After treatment with oral contraceptives, SHBG, INS, TESTO, and GLU (AUC > 0.6) were identified as effective markers for evaluating treatment outcomes. Both models successfully predicted the treatment efficacy in PCOS patients receiving oral contraceptives (AUC > 0.6) (**Figures 3A, B**).

### 3.4 Risk regression analysis identifies metabolic markers for prognostic risk in PCOS patients

**Figure 4:**
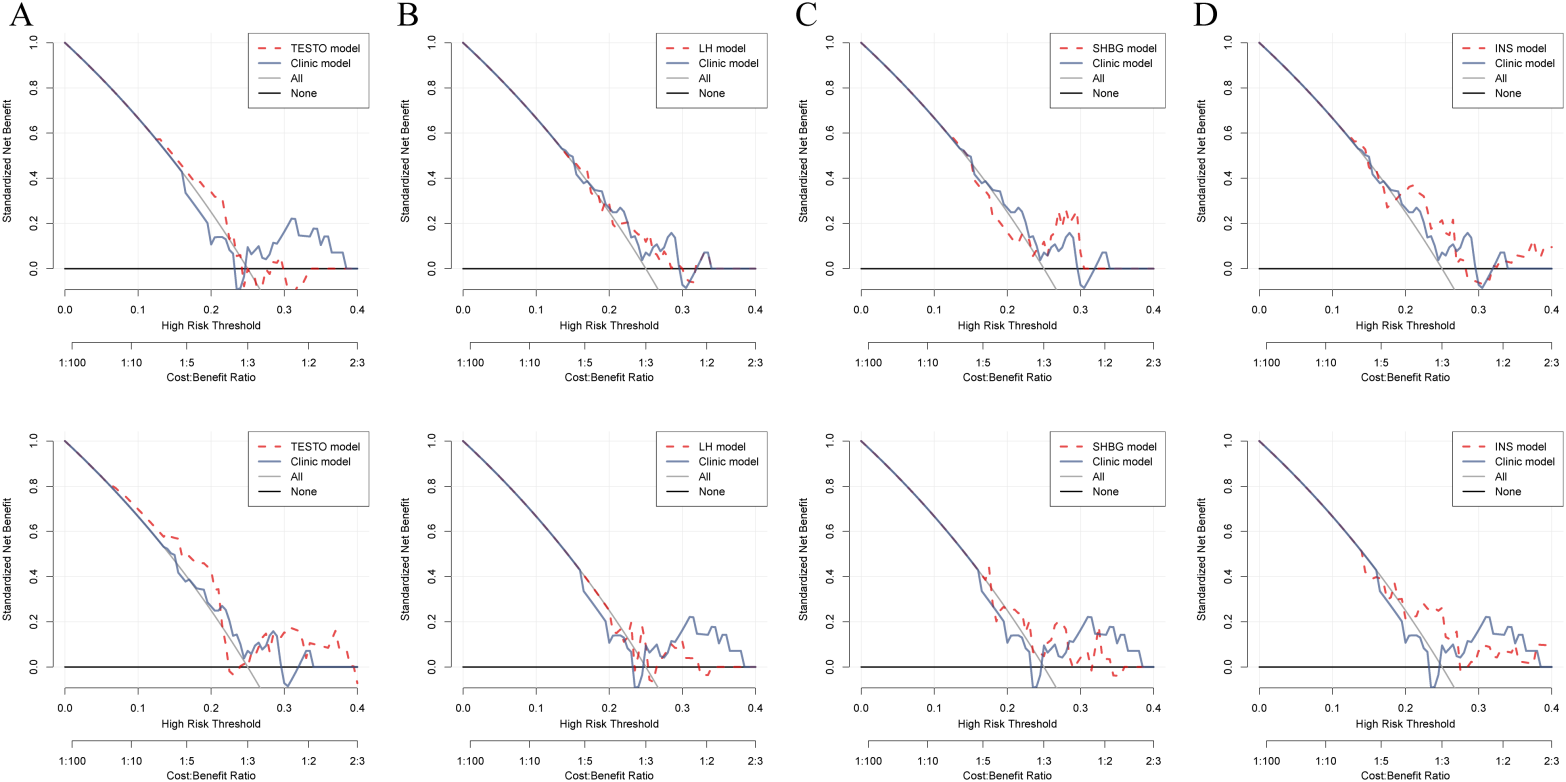
Comparison of the decision-making efficacy between metabolic biomarkers and existing clinical markers for PCOS patients before and after treatment. **(A–D)**. Decision curves for assessing treatment outcomes in PCOS patients, comparing TESTO, SHBG, LH, and INS with the established clinical biochemical marker LH/FSH.

TESTO is already recognized as a serological diagnostic marker for PCOS in clinical practice, as well as a prognostic indicator for adverse pregnancy outcomes in PCOS patients (32, 33). Using TESTO as a risk indicator in regression analysis for PCOS (34). Univariate regression analysis indicated that SHBG serves as a protective factor for PCOS (HR = 1.01, 95% CI: 1.01-1.02, *P* < 0.0001) (**Supplementary Table S3**). Multivariate regression analysis further revealed that FSH, LH, and LH/FSH are risk factors for PCOS (*P* < 0.05), while SHBG continues to be identified as a protective factor (HR = 1.01, 95% CI: 1.01-1.02, *P* < 0.0001) (**Supplementary Table S4**). These findings suggest that SHBG is a potential independent prognostic risk factor for PCOS. Additionally, the LH/FSH ratio is a significant clinical indicator for treatment decision-making and the risk of complications in PCOS, demonstrating high diagnostic efficacy (35, 36). By employing the LH/FSH ratio as a clinical decision parameter (Clinic model), we were able to compare the diagnostic efficacy of other metabolic markers associated with PCOS. DCA was performed to evaluate the clinical utility of TESTO, LH, SHBG, and INS in treatment decision-making. The results revealed that, prior to oral contraceptive treatment, SHBG and INS exhibited higher diagnostic efficacy for PCOS compared to the LH/FSH ratio. Following treatment, TESTO, SHBG, and INS demonstrated superior decision-making effectiveness over the LH/FSH ratio (**Figures 4A-D**). These findings highlight the high specificity of SHBG for the clinical diagnosis and treatment decision-making of PCOS, positioning it as a promising novel biomarker for the condition.

### 3.5 The association of SHBG with complications and adverse outcomes in PCOS

Univariate regression analysis of the association between SHBG and pregnancy outcomes, folate metabolism status, and embryo transfer outcomes in PCOS patients before and after treatment revealed that SHBG is an independent risk factor for pregnancy outcomes, folate metabolism disorders, and overall pregnancy outcomes prior to treatment (HR: 1.01, 95% CI: 1.01-1.07, *P* = 0.004; HR: 1.02, 95% CI: 1.01-1.04, *P* = 0.008; HR: 1.03, 95% CI: 1.01-1.06, *P* = 0.004) (**Supplementary Table S5**). These findings indicate that SHBG has significant diagnostic value for the prognosis of PCOS patients before treatment, although its relevance diminishes post-treatment. This suggests that SHBG may serve as a potential therapeutic target for PCOS.

### 3.6 Comparison of the decision-making efficacy between common clinical indicators for PCOS and SHBG

**Figure 5:**
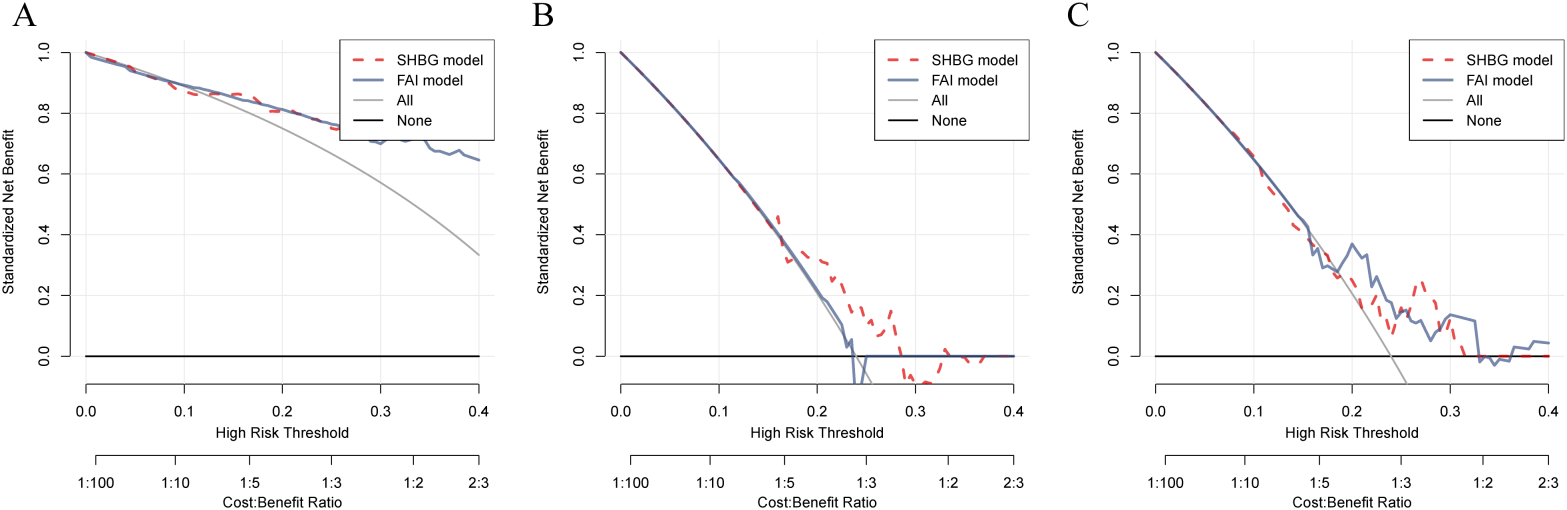
Comparison of the diagnostic capabilities of SHBG and FAI in PCOS patients before and after treatment, as well as in relation to pregnancy outcomes. **(A–C)**. Predictive efficacy of SHBG and FAI for PCOS patients before and after treatment and their pregnancy outcomes; the red dashed line represents the decision curve for SHBG, while the gray solid line represents the decision curve for FAI.

FAI is a biochemical marker for high-risk complications and adverse reproductive outcomes in clinical PCOS, demonstrating significant clinical value (37, 38). The DCA indicates that both SHBG and FAI exhibit comparable diagnostic capabilities for assessing outcomes after oral contraceptive treatment and predicting reproductive outcomes in PCOS. However, prior to treatment, SHBG demonstrated significantly superior clinical decision-making ability compared to FAI (*P* = 0.042) (**Figures 5A–C**, **Supplementary Table S6**).

### 3.7 Causal effects between SHBG and genetic plasma proteins in PCOS

Through two-sample bidirectional MR analysis, filtering for IVW *P-values* > 0.05 identified GCNT2, PIGN, KREMEN1, GCDH, CD93, CCDC77, and HSD17B13 as genetic risk proteins associated with PCOS and SHBG levels (**Supplementary Figure S2**). Notably, CD93 (OR: 1.138, 95% CI: 1.024-1.265, *P* = 0.016), GCNT2 (OR: 1.177, 95% CI: 1.058-1.310, *P* = 0.003), HSD17B13 (OR: 1.134, 95% CI: 1.024-1.255, *P* = 0.015), and PIGN (OR: 1.097, 95% CI: 1.014-1.186, *P* = 0.021) showed a strong association with the risk of PCOS. In contrast, CCDC77, GCDH, and KREMEN1 were identified as protective factors for the condition (**Supplementary Figure S4**). Sensitivity analyses using the leave-one-out method, along with tests for horizontal pleiotropy, indicated no evidence of genetic pleiotropy, horizontal diversity or heterogeneity affecting the results (**Supplementary Figures S6, S7**). Additionally, results from MR-Egger and MR-PRESSO further corroborated these findings. In the GSE87435 cohort, the risk plasma proteins were assessed, yielding a model AUC of 0.931 (95% CI: 0.778-1.000). The AUCs for CD93, GCDH, GCNT2, and PIGN were 0.653, 0.750, 0.847, and 0.569, respectively (**Supplementary Figure S3A**). Analysis of the expression levels of these risk proteins revealed significant differences in the mRNA expression of GCDH and GCNT2 between PCOS and normal ovarian tissues (*P* < 0.05) (**Supplementary Figure S3C**). These findings suggest that SHBG is likely to influence the expression levels of GCNT2, thereby mediating adverse prognoses in PCOS. All risk proteins are located outside the sex chromosomes, with GCNT2, GCDH, PIGN, and HSD17B13 situated on chromosomes 6, 19, 18, and 4, respectively (**Supplementary Figure S5B**).

### 3.8 GCNT2 regulates PI3K/AKT signaling in PCOS

**Figure 6:**
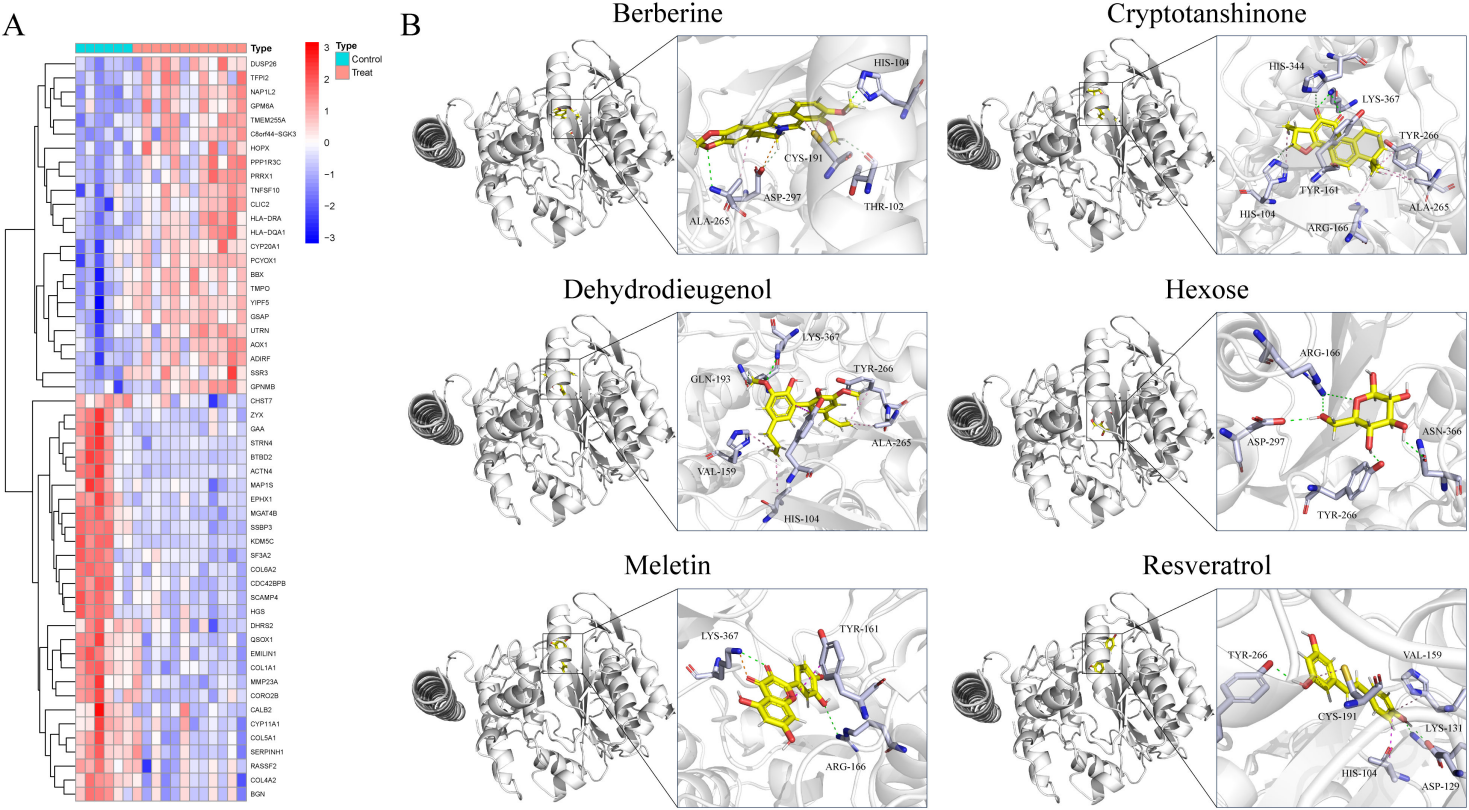
Identification of downstream regulatory targets of GCNT2 and virtual screening of naturally occurring small molecule compounds. **(A)** Downstream factors and co-expressed genes regulated by GCNT2 among DEGs in PCOS and normal ovarian tissues. **(B)** 3D docking model of candidate small molecule ligands with the GCNT2 protein receptor. The stick model illustrates the interactions of the protein side chain amino acids, with green dashed lines representing hydrogen bonds, light green dashed lines indicating carbon-hydrogen interactions, and pink dashed lines denoting π-π stacking, alkyl interactions, and π-alkyl interactions.

In the GSE87435 cohort, an integrative transcriptomic analysis based on GCNT2 expression levels identified 53 differentially expressed genes (DEGs) associated with variations in GCNT2 expression (*logFC* > 1, *P* < 0.05) (**Figure 6A**). PPI analysis revealed that COL1A1, COL4A2, COL5A1, and COL6A2 are central genes within the network (**Supplementary Figure S5A**). GO enrichment analysis indicated that GCNT2 is linked to collagen synthesis, the composition of ECM structural components, and connective tissue development. Furthermore, KEGG analysis demonstrated that GCNT2 positively regulates several signaling pathways, including ECM-receptor interaction, the AGE-RAGE signaling pathway in diabetic complications, and the PI3K-Akt signaling pathway (**Supplementary Figures S3B, C**). Notably, COL1A1, COL4A2, and COL6A2 were identified as key downstream factors of GCNT2 that modulate the PI3K-Akt signaling pathway.

### 3.9 Virtual screening of natural compounds targeting GCNT2

Through screening a natural product compound library, we identified 76 candidate molecules that target GCNT2, selecting the top six lead compounds for further analysis. Vina docking results indicated that *Hexose*, *Meletin*, *Resveratrol*, *Cryptotanshinone*, *Dehydrodieugenol*, and *Berberine* all exhibited specific binding to GCNT2 with relatively low binding energies of -4.7, -7.6, -6.9, -8.0, -7.0, and -8.0 kcal/mol, respectively (**Table 2**). CDOCKER analysis revealed that *Meletin*, *Resveratrol*, *Cryptotanshinone*, and berberine demonstrated low interaction energies and stable binding in both the CHARMM36 force field and physiological aqueous environments (**Table 2**). Notably, *Meletin* and *Cryptotanshinone* achieved LibdockScores > 100, indicating exceptional binding affinity and target specificity. The ligand interactions with GCNT2 primarily rely on hydrogen bonding, with *Cryptotanshinone* emerging as the most promising candidate for GCNT2-targeted therapy. It forms stable hydrogen bonds with key side chain amino acid residues HIS-344, LYS-367, HIS-104, and TYR-161 (**Figure 6B**). Comparative analysis highlighted that the side chain residues ALA-265, ARG-166, HIS-104, and TYR-266 of GCNT2 exhibit high druggability and low molecular binding energies, with their free hydroxyl and carbonyl groups capable of forming stable hydrogen bonds with the lead compounds.

**Table 2:** Molecular docking screening of potential drugs targeting GCNT2 for PCOS.

Candidates	LibdockScore	CDOCKER_energy	Interaction_energy	Vina score
*Hexose*	65.942	-9.077	-13.472	-4.7
*Meletin*	105.018	-34.196	-40.543	-7.6
*Resveratrol*	78.600	-24.433	-34.118	-6.9
*Cryptotanshinone*	109.774	-37.819	-41.233	-8.0
*Berberine*	85.994	-20.259	-35.629	-8.0
*Dehydrodieugenol*	97.444	-10.174	-42.314	-7.0

### 3.10 Pharmacokinetic and toxicokinetic analysis of *Cryptotanshinone*

**Figure 7:**
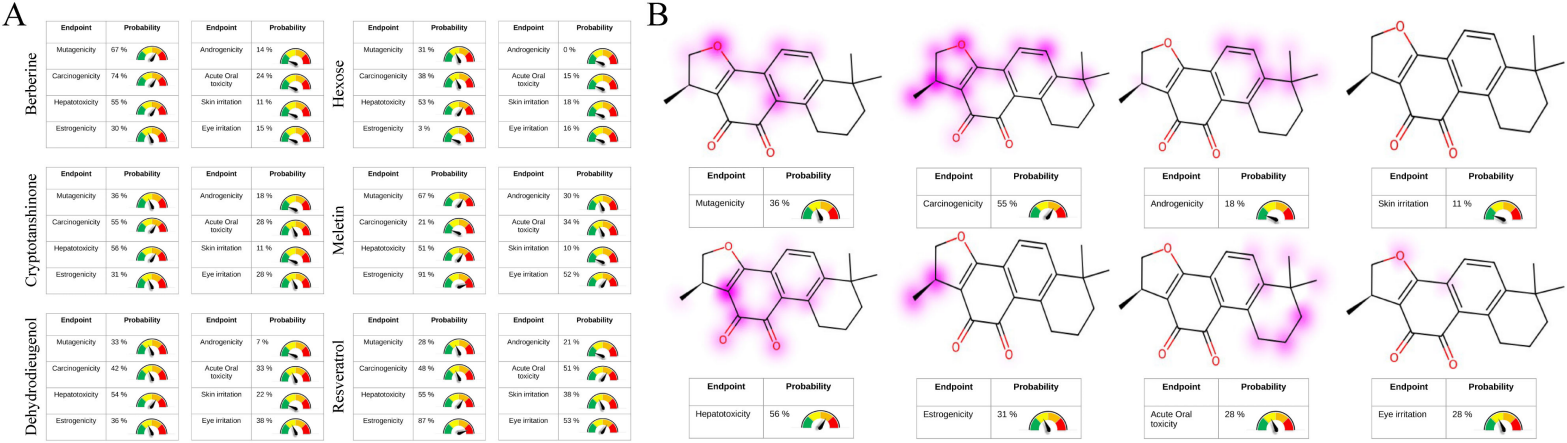
Toxicological properties analysis of candidate compounds and toxicophoric group staining. **(A)** Prediction of the candidate compounds’ structural properties related to hepatotoxicity, neurotoxicity, mutagenicity, hormonal toxicity, strong irritancy, and acute oral toxicity. **(B)** Identification of potential functional groups in the backbone structure of Cryptotanshinone associated with hepatotoxicity, neurotoxicity, mutagenicity, hormonal toxicity, strong irritancy, and acute oral toxicity.

The analysis of ADME properties *in vivo* indicated that all candidate compounds comply with Lipinski’s rule of five, demonstrating high synthetic accessibility (< 5) and drug-likeness (> 50%). With the exception of hexose, all compounds exhibited favorable gastrointestinal absorption. *Resveratrol*, *Cryptotanshinone*, *Berberine*, and *Dehydrodieugenol* showed high permeability across the blood-brain barrier (BBB) and displayed significant inhibition of cytochrome P450 enzymes, making them less susceptible to rapid degradation. Notably, resveratrol and *Dehydrodieugenol* are non-P-glycoprotein substrates, which reduces the degradation of the drugs during transport. All candidate compounds exhibited high solubility in both aqueous and lipid environments, with *Cryptotanshinone* demonstrating low molecular polarity and flexibility, suggesting that its pharmacophore structure is stable (**Supplementary Figures S8A–F**). Toxicokinetic parameters and functional group analyses revealed that *Resveratrol*, *Berberine*, *Dehydrodieugenol*, and *Meletin* possess considerable hepatotoxicity, neurotoxicity, mutagenicity, hormonal toxicity, strong irritancy, and acute oral toxicity (> 50%). In contrast, *Cryptotanshinone* and hexose exhibited lower biological toxicity (**Figure 7A**). These findings suggest that *Cryptotanshinone* is a promising candidate for targeting GCNT2 as a low-toxicity, high-efficacy natural compound for the treatment of PCOS. Toxicological functional group analysis indicated no significant toxicological risks associated with the core structure of *Cryptotanshinone*, further supporting its safety profile (**Figure 7B**).

### 3.11 Prediction of the direct effects of *Cryptotanshinone* on the PI3K/Akt signaling pathway

Through molecular docking studies, we investigated the interaction between *Cryptotanshinone* and the core proteins regulated by GCNT2 in the PI3K/Akt pathway, specifically COL1A1, COL4A2, and COL6A2. The results indicated that *Cryptotanshinone* can form stable complexes with COL1A1 and COL6A2, exhibiting low binding energies of -9.1 and -9.2 kcal/mol, respectively, along with high target specificity (LibdockScore > 100) (**Supplementary Table S7**). These interactions are primarily maintained through hydrogen bonding (**Supplementary Figures S10A–C**). Given that PCOS patients require long-term medication, we also assessed the palatability of the candidate compounds to enhance patient compliance. Predictions indicated that hexose has a 92% probability of being bitter, meletin 72%, resveratrol 68%, while *Cryptotanshinone* shows a 93% probability of being sweet. Berberine is predicted to be bitter with 100% confidence, and dehydrodieugenol with 91% (**Supplementary Figures S9A–F**). Consequently, these findings suggest that patients are likely to have good adherence to *Cryptotanshinone* due to its favorable taste profile.

### 3.12 GCNT2/SHBG axis modifies PI3K/Akt signaling by N-glycosylation

**Figure 8:**
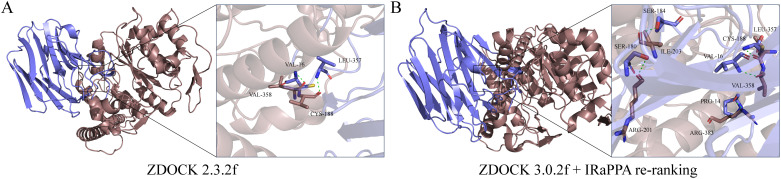
Revealing the N-glycosylation modification of SHBG protein by GCNT2 using FFT algorithms and deep learning models. **(A, B)** Construction of the GCNT2-SHBG complex using ZDOCK 2.3.2f and ZDOCK 3.0.2f with IRaPPA re-ranking algorithms. The stick model represents the side chain amino acid residues of the proteins, with green dashed lines indicating hydrogen bond interactions. The brown protein represents the receptor GCNT2, while the blue protein represents the ligand SHBG.

**Figure 9:**
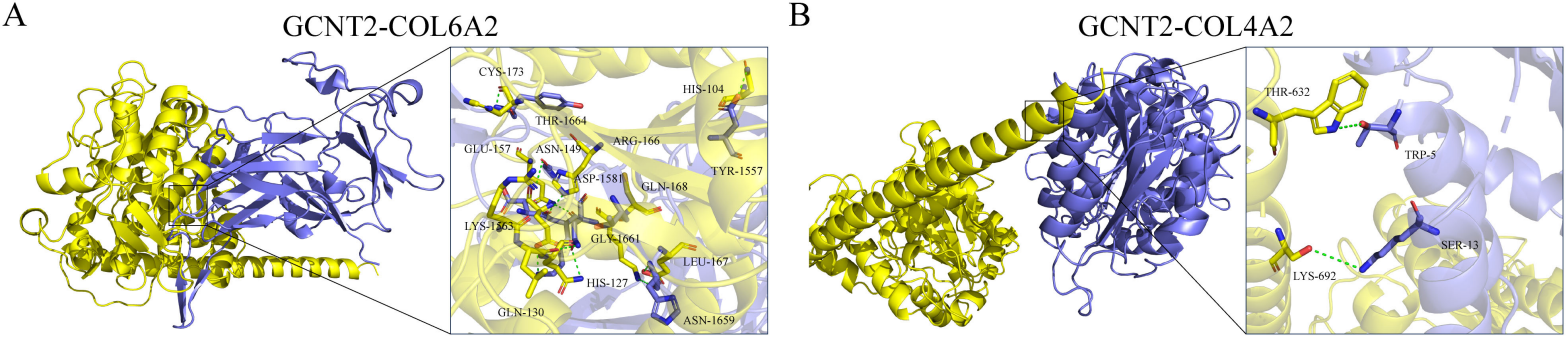
GCNT2 mediates N-glycosylation modifications in the PI3K/Akt signaling pathway. **(A, B)** 3D docking model of the protein-protein interactions between GCNT2 and COL6A2, as well as COL4A2. The yellow protein represents the receptor GCNT2, while the blue proteins represent the ligands COL4A2 and COL6A2. The stick model illustrates the side chain amino acid residues, with green dashed lines indicating hydrogen bond interactions.

Using ZDOCK 2.3.2f and ZDOCK 3.0.2f with IRaPPA re-ranking techniques, we investigated the N-glycosylation modification of SHBG by GCNT2 and identified the relevant binding sites. The results demonstrated that GCNT2 interacts with SHBG through the side chain residues CYS-188 and VAL-358, targeting the protein residues LEU-357 and VAL-16 for N-glycosylation modification. Following this modification, SHBG is transformed into a glycoprotein, which aligns with its role in androgen transport (**Figures 8A, B**). GCNT2 mediates glycosylation modifications on the core protein COL4A2 of the PI3K/Akt signaling pathway through the side chain residues THR-632 and LYS-692, specifically targeting the side chain residues TRP-5 and SER-13 of the A chain. Additionally, GCNT2 regulates the PI3K/Akt signaling pathway by N-glycosylating several critical residues on the B and C chains of COL6A2, including LYS-1563 (GLN-130, GLU-157, HIS-127), ASN-1659 (GLN-168, LEU-167), and HIS-1666 (LYS-155, GLU-175) (**Figures 9A, B**).

## 4 Discussion

PCOS is a common endocrine disorder that affects 6-10% of women of reproductive age, manifesting as irregular menstrual cycles, hyperandrogenism, and the presence of polycystic ovaries (7, 39). The pathophysiology of PCOS is complex and multifactorial, involving intricate interactions between genetic, environmental, and metabolic factors that give rise to its diverse clinical manifestations. Current diagnostic frameworks, largely based on the Rotterdam criteria, frequently lead to underdiagnosis or misdiagnosis, underscoring the syndrome’s inherent heterogeneity and the challenges in its accurate identification (1, 8). Existing biomarkers, such as elevated LH and TESTO, exhibit limitations in sensitivity and specificity (40–42). Furthermore, conventional treatment modalities, including hormonal contraceptives and insulin sensitizers, may fail to adequately address the underlying metabolic disturbances associated with PCOS (43). Recent research has shed light on the intricate molecular mechanisms underlying PCOS, particularly the pivotal roles of the PI3K/Akt signaling pathway and glycosylation processes (44, 45). These insights highlight the urgent need for innovative diagnostic and therapeutic strategies that not only enhance our understanding of PCOS but also pave the way for personalized treatment approaches tailored to individual patient profiles.

The GCNT2/SHBG axis plays a crucial role in the hormonal regulation and metabolic health of women with PCOS. Recent studies have shown that variations in GCNT2 expression significantly influence levels of SHBG, which in turn modulates the bioavailability of androgens (46). Elevated levels of androgens, often observed in PCOS patients, are associated with numerous metabolic disturbances, including insulin resistance and dyslipidemia (47). This regulatory axis not only underscores the pathophysiological links between hyperandrogenism and metabolic dysfunction but also positions GCNT2 as a potential therapeutic target for restoring metabolic balance (48). Our findings demonstrate that variations in GCNT2 expression significantly influence SHBG levels, thereby modulating the bioavailability of androgens. Elevated androgen levels, commonly observed in PCOS patients, are closely linked to metabolic disturbances such as insulin resistance and dyslipidemia. By elucidating the mechanisms through which GCNT2 regulates SHBG and subsequent androgen dynamics, we provide critical insights into the hormonal dysregulation that underpins PCOS pathophysiology.

Furthermore, alterations in N-glycosylation patterns associated with GCNT2 have significant implications for metabolic dysregulation in PCOS. Our research demonstrates that changes in glycosylation affect insulin signaling pathways, exacerbating insulin resistance: a hallmark of PCOS (49). The aberrant glycosylation of proteins can disrupt normal cellular functions, leading to impaired glucose metabolism and increased adiposity (50). By integrating the roles of the GCNT2/SHBG axis and N-glycosylation, we reveal a complex interplay that contributes to the metabolic phenotype of PCOS. These findings suggest that targeting glycosylation processes may offer new avenues for therapeutic intervention, potentially mitigating the metabolic complications often faced by women with PCOS. Overall, our study emphasizes the importance of the GCNT2/SHBG axis and N-glycosylation in understanding and addressing the multifaceted nature of PCOS, paving the way for more effective, personalized treatment strategies.

The PI3K/Akt signaling pathway plays a crucial role in the metabolic and reproductive dysfunctions associated with PCOS. Our findings indicate that dysregulation of this pathway significantly contributes to insulin resistance and impaired ovarian function, both of which are hallmark features of PCOS. Specifically, we observed that aberrant activation of the PI3K/Akt pathway is linked to increased androgen production and disrupted follicle development, further aggravating the clinical manifestations of the syndrome (51). This connection underscores the pathway’s critical role in bridging metabolic disturbances and hormonal imbalances, suggesting that targeted interventions aimed at modulating this signaling cascade may offer promising therapeutic options for managing PCOS-related complications. Importantly, our research highlights how GCNT2 regulates the PI3K/Akt signaling pathway through N-glycosylation modifications. Changes in GCNT2 expression can alter the glycosylation patterns of key signaling molecules, impacting their function and stability. This modification has profound implications for insulin signaling and ovarian responsiveness, further linking GCNT2 to the metabolic dysfunction observed in PCOS. By influencing the PI3K/Akt pathway *via* N-glycosylation, GCNT2 serves as a critical mediator that not only affects androgen levels but also addresses the underlying insulin resistance that characterizes the syndrome. This intricate interplay suggests that targeting GCNT2 could provide a dual therapeutic strategy: enhancing ovarian function while simultaneously improving metabolic health. Therefore, understanding the role of GCNT2 in modulating the PI3K/Akt pathway through glycosylation presents an exciting avenue for developing novel interventions aimed at alleviating the multifaceted challenges faced by women with PCOS (52).

Natural products have demonstrated the advantages of high efficiency, low toxicity, and easy synthesis in the treatment of diseases (53). *Cryptotanshinone*, bioactive compound derived from *Salvia miltiorrhiza*, exhibits considerable therapeutic potential for various metabolic and endocrine disorders, particularly PCOS. Recent research demonstrates that *Cryptotanshinone* enhances insulin sensitivity by activating the PI3K/Akt signaling pathway, a crucial mechanism often disrupted in PCOS, facilitating improved glucose uptake in peripheral tissues (13). Additionally, this compound displays anti-androgenic effects, evidenced by its ability to reduce androgen production in ovarian cells, thereby alleviating hyperandrogenism symptoms commonly experienced by affected women. *Cryptotanshinone* possesses anti-inflammatory properties, effectively downregulating pro-inflammatory cytokines, which play a significant role in the pathophysiology of PCOS (54). Our findings also indicate that *Cryptotanshinone* may modulate glycosylation processes, particularly influencing the GCNT2/SHBG axis, further enhancing metabolic health (55). In preclinical studies, treatment with *Cryptotanshinone* restored normal ovarian morphology and function, indicating its potential to improve fertility outcomes in women with PCOS (56).

From a pharmacokinetic perspective, *Cryptotanshinone* has shown favorable absorption and distribution characteristics, with minimal toxicity reported, making it a promising candidate for clinical applications (57). Molecular docking studies demonstrate that *Cryptotanshinone* exhibits strong interactions with key proteins implicated in PCOS-related pathways, offering mechanistic insights into its therapeutic potential. These findings suggest that *Cryptotanshinone* effectively targets hormonal imbalances and metabolic dysfunction, providing a dual therapeutic approach that enhances ovarian function and improves metabolic health. Further exploration of its synergistic effects with other therapeutic agents holds promise for developing integrated treatment strategies, advancing the management of PCOS (58).

Future research should focus on validating *Cryptotanshinone* associated biomarkers and therapeutic candidates to enhance their clinical applicability in PCOS management. Longitudinal studies are crucial to assess the long-term efficacy, safety, and potential cumulative effects of *Cryptotanshinone* and related natural compounds.

## 5 Limitations

While our study highlights the therapeutic potential of *Cryptotanshinone* in PCOS management through modulation of the GCNT2/SHBG axis, several limitations must be acknowledged. First, the preclinical nature of our research, particularly the reliance on MR analyses, necessitates caution in extrapolating these results to clinical settings. Although MR provides robust evidence for causal inference, potential biases such as horizontal pleiotropy or population stratification may confound the observed genetic associations. Future studies should incorporate sensitivity analyses and multivariable MR approaches to address these limitations.

Our sample size, while sufficient for initial analyses, may limit the generalizability of the results, particularly in capturing the full spectrum of PCOS heterogeneity. Larger, multi-center cohorts with diverse ethnic backgrounds are needed to confirm these findings. Additionally, the study’s focus on the GCNT2/SHBG axis may not fully capture the complexity of PCOS pathophysiology, which involves intricate metabolic, endocrine, and inflammatory interactions.

Individual variations in treatment response, influenced by genetic, epigenetic, and lifestyle factors, were not extensively addressed. Future research should incorporate pharmacogenomic analyses and longitudinal designs to develop personalized treatment strategies. Moreover, while molecular docking and ADME analyses suggest *Cryptotanshinone* as a promising candidate, experimental validation in preclinical models and clinical trials is essential to translate these findings into therapeutic applications.

Finally, reliance on existing data may overlook emerging biomarkers or pathways relevant to PCOS. Future studies should explore novel therapeutic targets and evaluate synergistic effects of *Cryptotanshinone* with existing therapies. Despite these limitations, our findings provide a robust foundation for further investigation into *Cryptotanshinone* as the potential therapeutic agent, particularly in targeting the GCNT2 pathway to improve PCOS management. We hope these insights will inspire future research to address the identified gaps and advance personalized treatments for PCOS.

## 6 Conclusion

**Figure 10:**
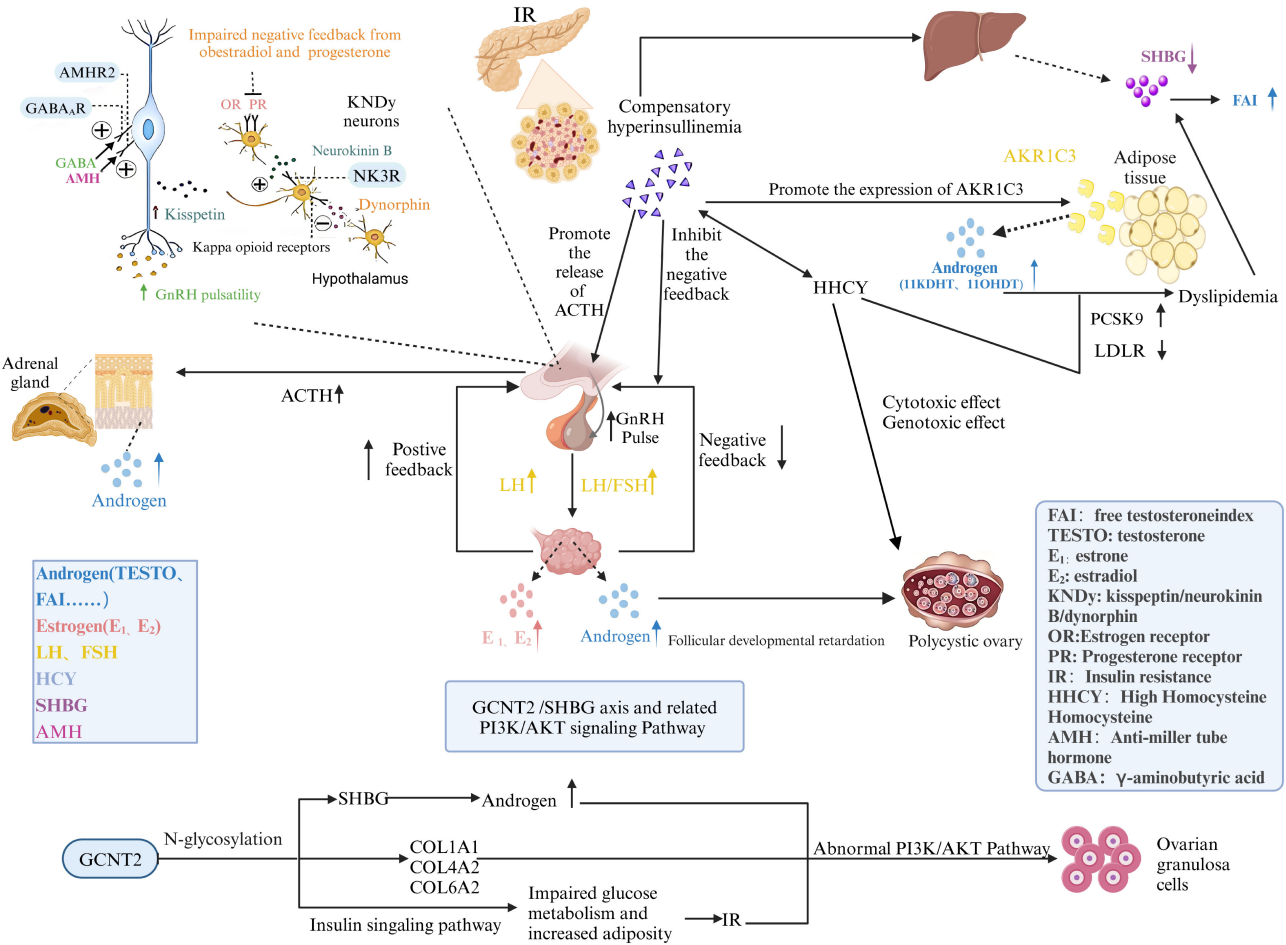
A meta-analysis depicting the mechanisms underlying the onset and progression of PCOS.

This study elucidates the critical role of the GCNT2/SHBG axis in regulating N-glycosylation and the PI3K/Akt signaling pathways, offering novel insights into PCOS pathophysiology (**Figure 10**). Using a well-defined patient cohort and MR, we established causal relationships that underscore GCNT2 as a key biomarker. GCNT2-mediated alterations in N-glycosylation modulate SHBG levels, insulin sensitivity, and ovarian function, directly linking metabolic disruptions to PCOS clinical manifestations. Moreover, the dysregulation of the PI3K/Akt pathway emerged as a central mechanism translating these metabolic changes into endocrine dysfunction, highlighting a sophisticated regulatory network governing metabolic and reproductive health.

Advanced machine learning techniques identified novel biomarkers, enhancing diagnostic precision and paving the way for personalized treatment strategies. Protein-protein docking studies revealed *Cryptotanshinone* as a promising therapeutic candidate, with potential to modulate the GCNT2/SHBG axis and influence the PI3K/Akt pathway. This integrative approach deepens our understanding of PCOS molecular mechanisms and underscores the therapeutic potential of targeting these pathways. Collectively, this research establishes a foundation for precision medicine in PCOS, aiming to refine treatments and improve patient outcomes.

## Data Availability

The original contributions presented in the study are included in the article/**Supplementary Material**, further inquiries can be directed to the corresponding author/s.

## Glossary

PCOS: Polycystic ovary syndrome
GCNT2: Glucosaminyl (N-acetyl) transferase 2
SHBG: Sex hormone-binding globulin
MR: Mendelian randomization
EE-CA: Estradiol-cyproterone acetate
AIDD: Artificial intelligence-driven drug design
WHR: Waist-to-hip ratio
FSH: Follicle-stimulating hormone
LH: Luteinizing hormone
TESTO: Total testosterone
FAI: Free testosterone
LDL-C: Low-density lipoprotein cholesterol
HDL-C: High-density lipoprotein cholesterol
TG: Triglycerides
total-C/CHOL: Total cholesterol
AMH: Anti-Müllerian Hormone
RF: Random forest
DCA: Decision curve analysis
GWAS: Genome-wide association study
pQTL: Protein quantitative trait loci
eQTL: Expression quantitative trait loci
LD: Linkage disequilibrium
SNPs: Single nucleotide polymorphisms
WR: Wald ratio
IV: Instrumental variable
IVW: Inverse variance weighting
OR: Odds ratios
95%CI: 95% confidence intervals
FDR: False discovery rate
ROC: Receiver operating characteristic
GO: Gene Ontology
KEGG: Kyoto Encyclopedia of Genes and Genomes
HTVS: High-throughput virtual screening
DS: Discovery Studio 2019
ADME: Absorption, distribution, metabolism and excretion
FFT: Fast fourier transform
HHCY: Hyperhomocysteinemia
INS: Insulin
DEGs: Differentially expression genes
BBB: Blood-brain barrier.
