# Supplementary material for: Blood biochemical landscape and new insights into clinical decision-making for polycystic ovary syndrome in Chinese women: a prospective cohort study
Source: Front Endocrinol (Lausanne). 2025 May 1;16:1534733. doi: 10.3389/fendo.2025.1534733 (PMC12078145; doi:10.3389/fendo.2025.1534733)

**Supplementary figures**

**Figure S1.** Machine learning training of potential biomarker models for PCOS. A, B. A cohort of 103 PCOS patients were screened for potential biomarkers of PCOS using LASSO regression and RF, respectively.


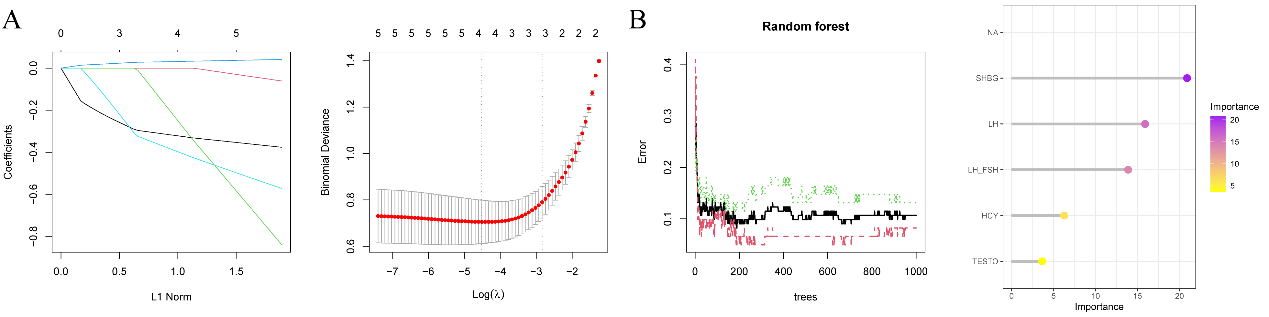


**Figure S2.** PCOS and SHBG common genetic association risk plasma proteins.


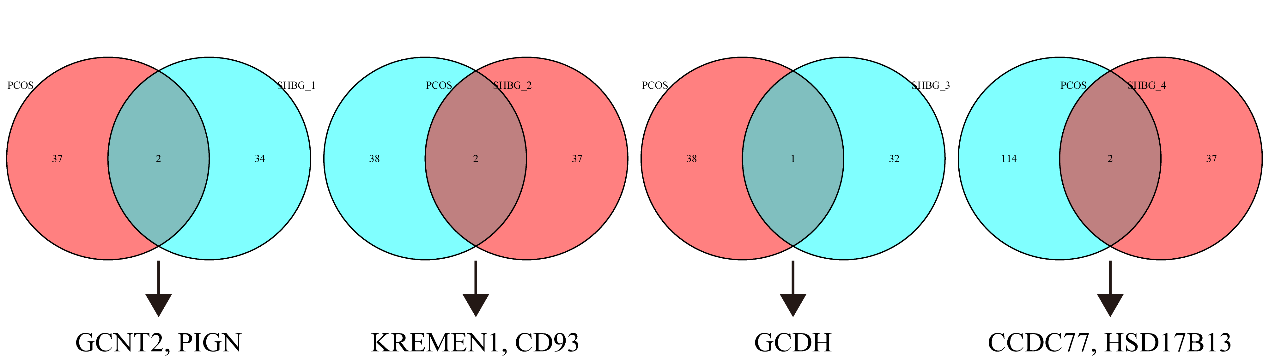


**Figure S3.** SHBG-mediated screening and functional analysis of plasma protein markers of PCOS risk. A. Specificity and diagnostic AUC curves of risk plasma protein markers in PCOS. B, C. GO and KEGG enrichment analyses were conducted to explore the signaling pathways regulated by GCNT2 in PCOS and to identify potential downstream molecular targets. D. mRNA expression levels of plasma protein markers in PCOS and normal ovarian tissues. *^*^P* < 0.05, *^**^P* < 0.01, *^***^P* < 0.001.


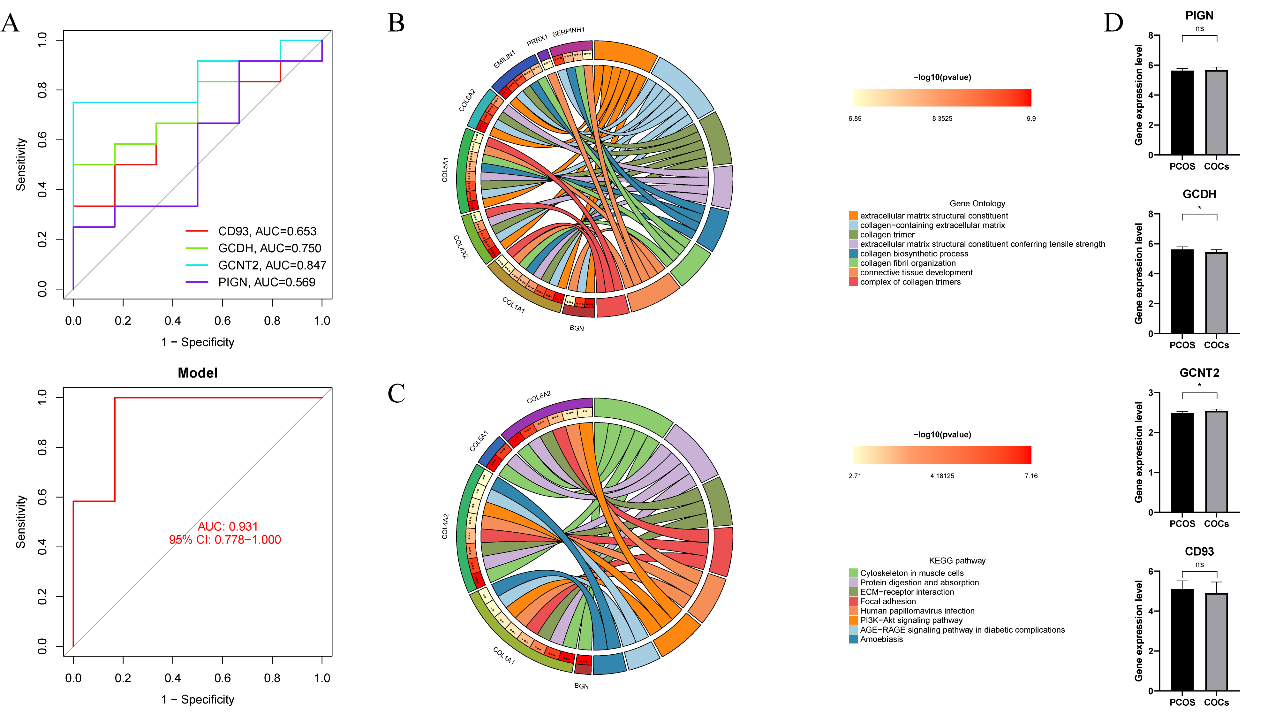


**Figure S4.** Two-sample bidirectional Mendelian randomization was used to investigate the causal associations between genetically shared SNPs for PCOS and SHBG, and plasma protein SNPs.


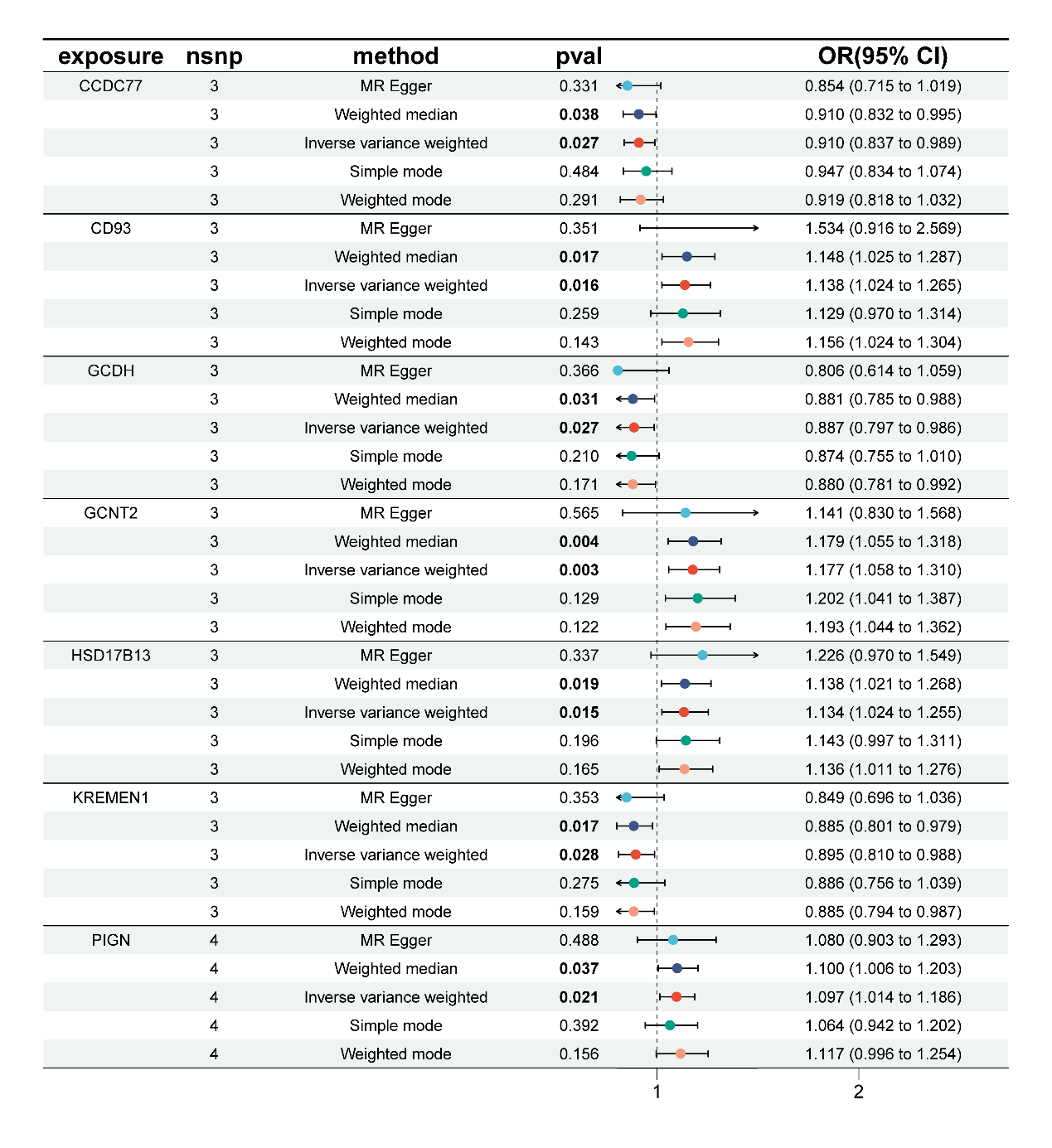


**Figure S5.** GCNT2 protein regulatory network and chromosomal co-localization of risk plasma proteins. A. Downstream molecular targets regulated by GCNT2 in PCOS and the co-expression gene PPI network diagram. B. Chromosomal co-localization analysis of PCOS-SHBG plasma protein markers.


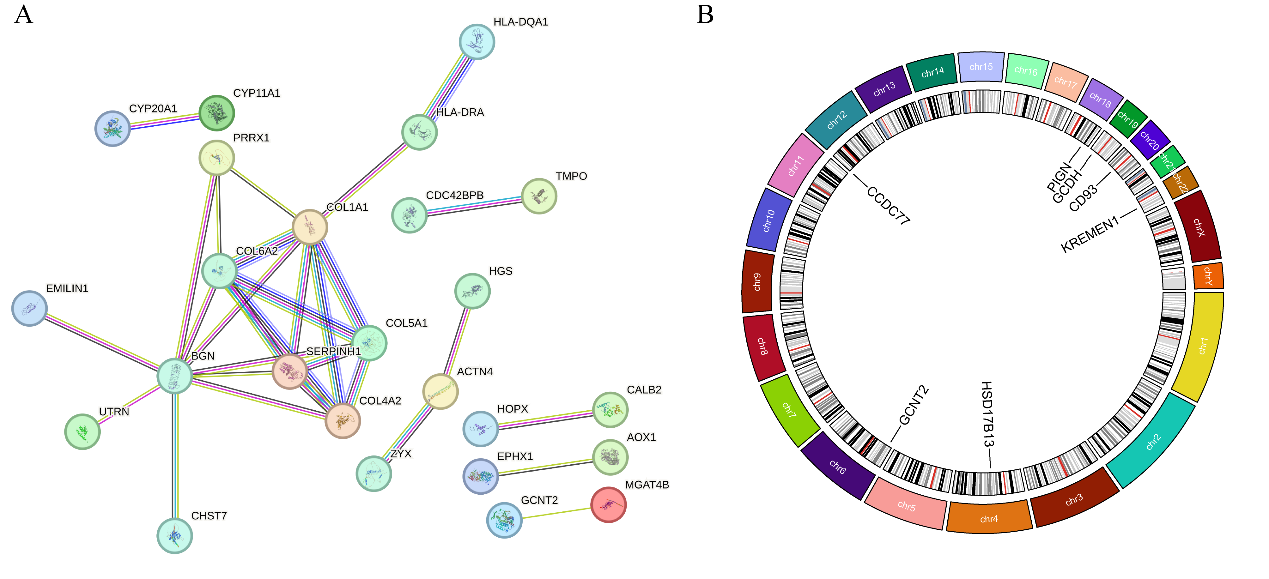


**Figure S6.** MR-Egger regression and funnel plot analyses were employed to assess the presence of horizontal pleiotropy and genetic pleiotropy in the genetic associations between PCOS and SHBG plasma protein markers. A. Forest plot of MR-Egger associated SNPs. B. Scatter plot for GWAS data analysis.


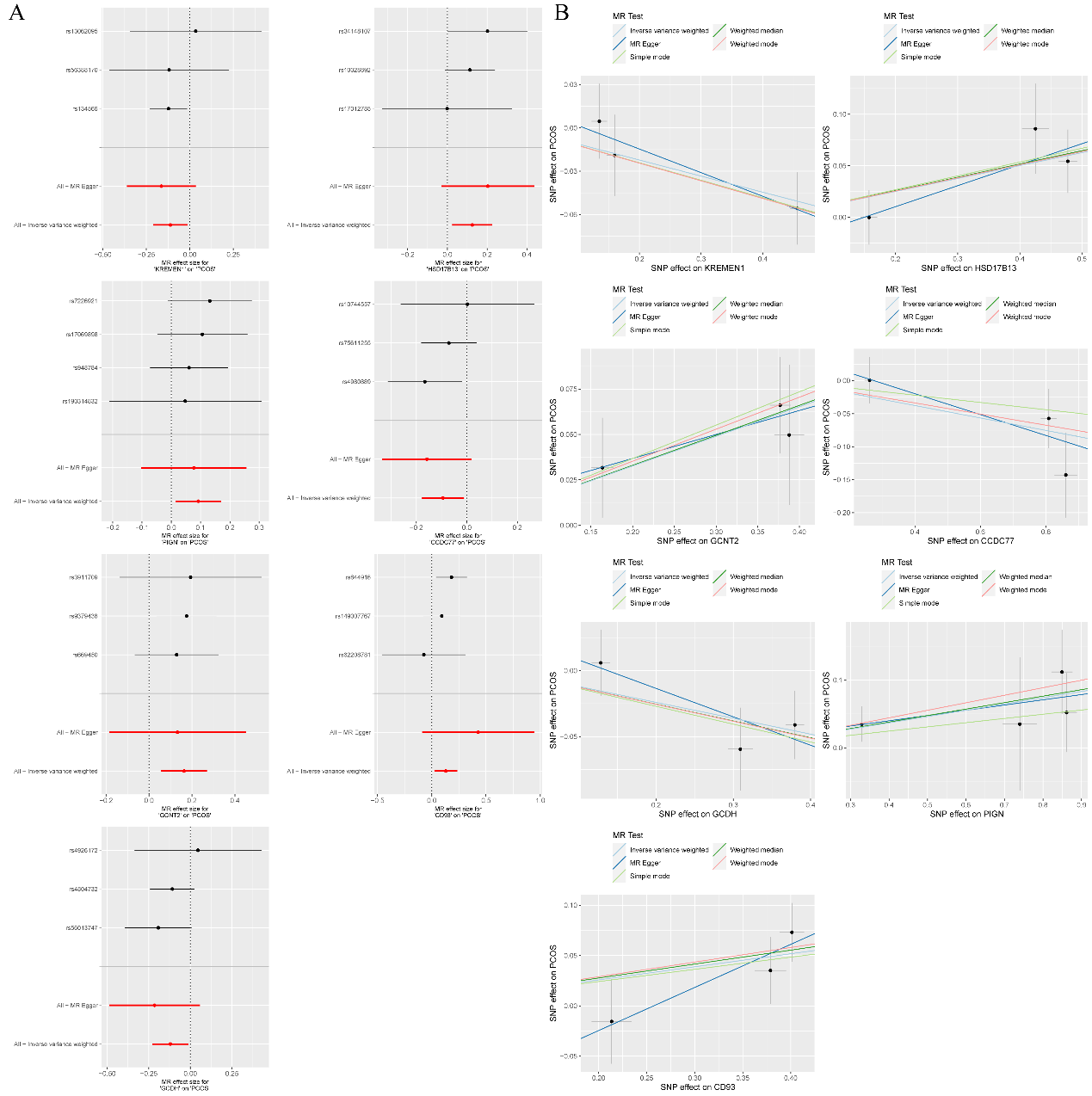


**Figure S7.** Leave-one-out sensitivity analysis and funnel plots were used to exclude heterogeneity in the genetic associations between PCOS and SHBG plasma protein markers. A. Funnel plot for heterogeneity assessment of the PCOS/SHBG plasma protein data. B. Leave-one-out sensitivity analysis was conducted to exclude SNP heterogeneity present in the genetic associations between PCOS and SHBG plasma protein markers.


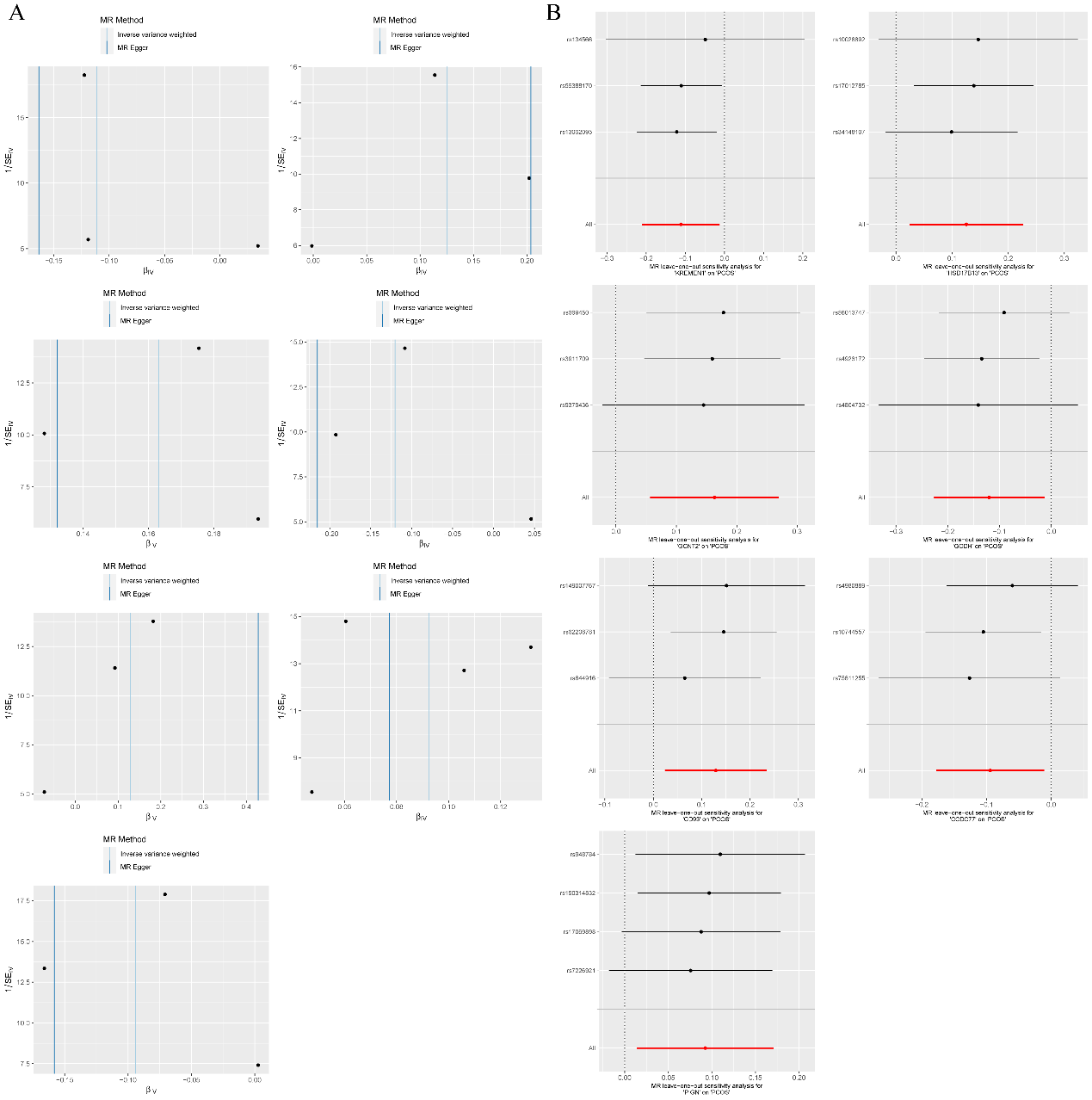


**Figure S8.** Pharmacokinetic properties analysis of candidate compounds. A-F. ADME property analysis of hexose, meletin, resveratrol, cryptotanshinone, berberine, and dehydrodieugenol in vivo.


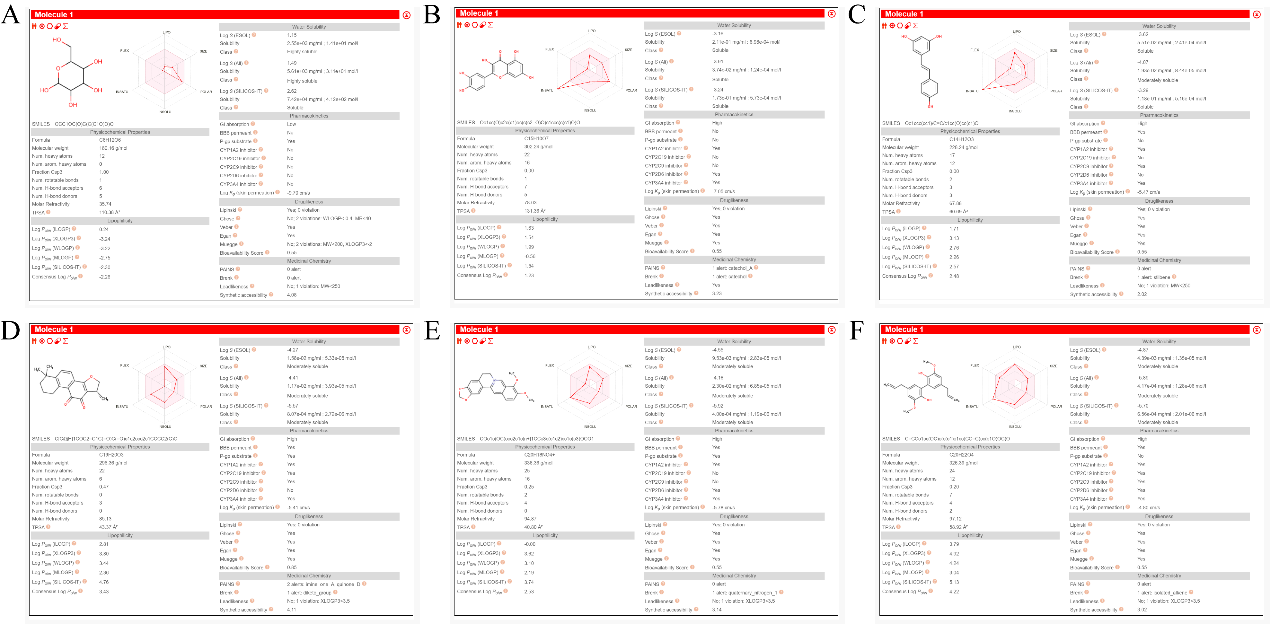


**Figure S9.** Analysis of patient compliance regarding candidate compounds for PCOS. A-F. Taste properties of hexose, meletin, resveratrol, cryptotanshinone, berberine, and dehydrodieugenol.


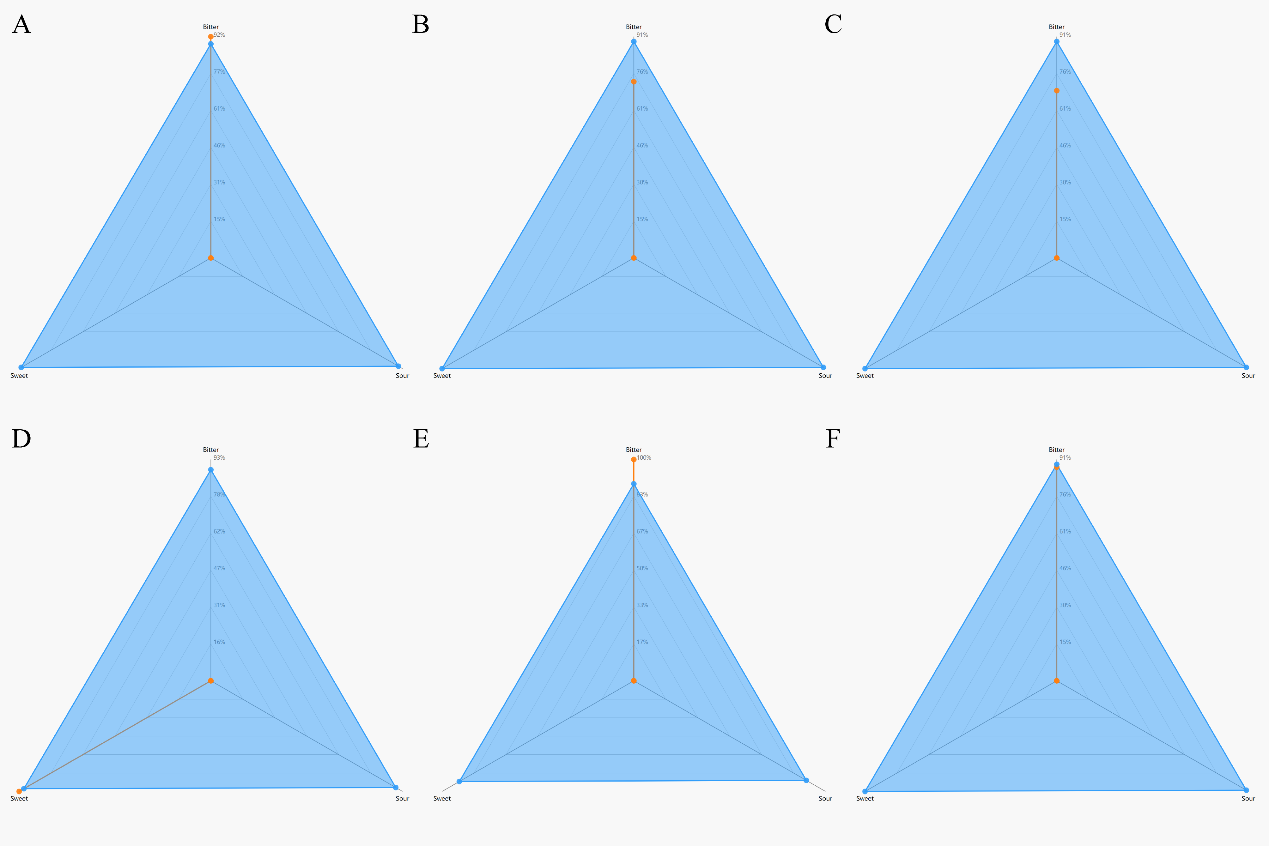


**Figure S10.** Cryptotanshinone targets key molecules in the PI3K/Akt signaling pathway. A-C. 3D binding mode diagram of cryptotanshinone with COL1A1, COL4A2, and COL6A2. The stick model represents the interactions of the protein side chain amino acids, with green dashed lines indicating hydrogen bonds, light green dashed lines representing carbon-hydrogen interactions, and pink dashed lines denoting π-π stacking, alkyl interactions, and π-alkyl interactions.


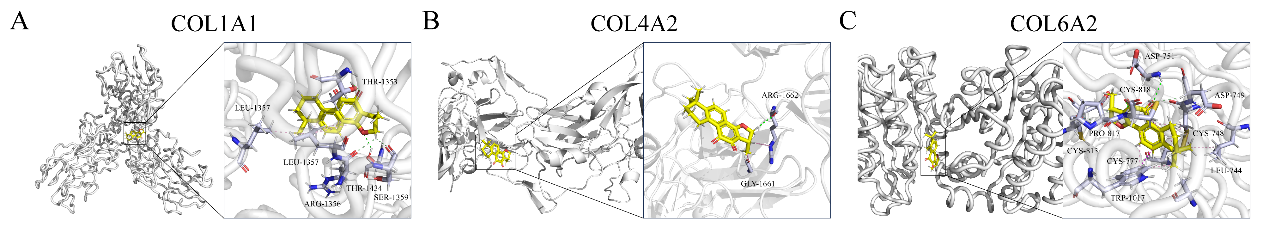

Supplement: Supplementary file 1 [file DataSheet1.docx]
