# Supplementary material for: Blood biochemical landscape and new insights into clinical decision-making for polycystic ovary syndrome in Chinese women: a prospective cohort study
Source: Front Endocrinol (Lausanne). 2025 May 1;16:1534733. doi: 10.3389/fendo.2025.1534733 (PMC12078145; doi:10.3389/fendo.2025.1534733)
Supplement: Supplementary file 2 [file Table1.docx]

**Supplementary tables**

Table S1. Clinical trait associations of SHBG indicators in PCOS patients at baseline and after treatment (R-Spearman linear regression & LASSO regression).

| Indicators | Samples | *R* | *P-value* |
| --- | --- | --- | --- |
| BMI (kg/m^2^) | 50 | -0.32 | 0.0013 |
| FSH (mIU/ml) | 82 | 0.09 | NS |
| LH (mIU/ml) | 84 | -0.17 | 0.03 |
| FSH/LH | 82 | -0.05 | *NS* |
| TESTO (ng/ml) | 97 | -0.24 | 0.0006 |
| GLU (mmol/L) | 88 | -0.20 | 0.0078 |
| INS (IU/ml) | 83 | -0.22 | 0.005 |
| HCY (μmol/L) | 76 | -0.32 | < 0.0001 |

Table S2. Clinical trait associations of SHBG indicators in PCOS patients at baseline and after treatment (R-Pearson linear regression & LASSO regression).

| Indicators | *R^2^* (Baseline/Treat) | *R* | *P-value* |
| --- | --- | --- | --- |
| BMI (kg/m^2^) | 0.14/0.14 | -0.34 | 0.00044 |
| FSH (mIU/ml) | 0.05/0.00 | 0.03 | NS |
| LH (mIU/ml) | 0.11/0.00 | -0.14 | NS |
| FSH/LH | 0.03/0.01 | -0.06 | NS |
| TESTO (ng/ml) | 0.00/0.02 | -0.25 | 0.0004 |
| GLU (mmol/L) | 0.01/0.05 | -0.20 | 0.0074 |
| INS (IU/ml) | 0.09/0.08 | -0.15 | NS |
| HCY (μmol/L) | 0.01/0.03 | -0.31 | < 0.0001 |

Table S3. Indicators of PCOS risk factors revealed by cox univariate analysis.

| Characteristics | Hazard.Ratio | CI_95_ | *P-value* |
| --- | --- | --- | --- |
| FSH (mIU/ml) | 0.89 | 0.74-1.07 | NS |
| LH (mIU/ml) | 0.94 | 0.88-1.00 | NS |
| LH/FSH | 0.99 | 0.86-1.01 | NS |
| SHBG | 1.01 | 1.01-1.02 | < 0.0001 |
| GLU (mmol/L) | 1.20 | 0.77-1.88 | NS |

Table S4. Indicators of PCOS risk factors revealed by multi-cox univariate analysis.

| Characteristics | Hazard.Ratio | CI_95_ | *P-value* |
| --- | --- | --- | --- |
| FSH (mIU/ml) | 1.50 | 1.03-2.18 | 0.036 |
| LH (mIU/ml) | 0.73 | 0.58-0.93 | 0.009 |
| LH/FSH | 5.99 | 1.44-24.95 | 0.014 |
| SHBG | 1.01 | 1.01-1.02 | < 0.0001 |
| GLU (mmol/L) | 1.72 | 0.95-3.10 | NS |

Table S5. Cox univariate analysis to test the efficacy of SHBG before and after treatment on pregnancy outcome, folate metabolism type, and embryo transfer modality.

| Characteristics | Hazard.Ratio | CI_95_ | *P-value* |
| --- | --- | --- | --- |
| Pregnancy | 1.01 | 1.01-1.07 | 0.004 |
|  | 1.00 | 0.99-1.01 | NS |
| Embryo transfer | 1.05 | 1.00-1.09 | NS |
|  | 1.01 | 1.00-1.03 | NS |
| Folate metabolism | 1.02 | 1.01-1.04 | 0.008 |
|  | 1.01 | 1.00-1.01 | NS |
| Outcome | 1.03 | 1.01-1.06 | 0.004 |
|  | 0.99 | 0.98-1.01 | NS |

Table S6. Comparison of the clinical significance of SHBG and FAI parametric diagnostic models.

| Characteristics | Hazard.Ratio | CI_95_ | *P-value* |
| --- | --- | --- | --- |
| Pregnancy (FAI) | 0.93 | 0.85-1.01 | NS |
|  | 0.98 | 0.86-1.13 | NS |
| Pregnancy (SHBG) | 0.99 | 0.97-1.01 | NS |
|  | 0.99 | 0.98-1.00 | 0.042 |
| Therapeutic (FAI) | 0.70 | 0.63-0.77 | < 0.0001 |
| Therapeutic (SHBG) | 1.01 | 1.01-1.02 | < 0.0001 |

Table S7. Molecular docking of cryptotanshinone with core proteins of the PI3K/AKT signaling pathway

| Protein | LibdockScore | CDOCKER_energy | Interaction_energy | Vina score |
| --- | --- | --- | --- | --- |
| COL1A1 | 129.846 | -59.344 | -62.274 | -9.1 |
| COL4A2 | 83.440 | -26.592 | -19.881 | -6.9 |
| COL6A2 | 166.952 | -55.869 | -38.665 | -9.2 |
